# Treatment landscape and burden of disease in metastatic castration-resistant prostate cancer: systematic and structured literature reviews

**DOI:** 10.3389/fonc.2023.1240864

**Published:** 2023-09-27

**Authors:** Darren Leaning, Gagandeep Kaur, Alicia K. Morgans, Ray Ghouse, Osvaldo Mirante, Simon Chowdhury

**Affiliations:** ^1^ Department of Radiology and Oncology, James Cook University Hospital, South Tees NHS Trust, Middlesbrough, United Kingdom; ^2^ Parexel Access Consulting, Parexel International, Mohali, Punjab, India; ^3^ Department of Medical Oncology, Dana-Farber Cancer Institute, Boston, MA, United States; ^4^ Advanced Accelerator Applications, a Novartis Company, Genève, Switzerland; ^5^ Department of Urological Cancer, Guy’s, King’s, and St. Thomas’ Hospitals, and Sarah Cannon Research Institute, London, United Kingdom

**Keywords:** metastatic castration-resistant prostate cancer, treatment landscape, treatment pattern, burden of disease, cost, economic burden

## Abstract

**Purpose:**

Metastatic castration-resistant prostate cancer (mCRPC) is a lethal disease that imposes a major burden on patients and healthcare systems. Three structured literature reviews (treatment guidelines, treatment landscape, and human/clinical/patient burden) and one systematic literature review (economic burden) were conducted to better understand the disease burden and unmet needs for patients with late-stage mCRPC, for whom optimal treatment options are unclear.

**Methods:**

Embase^®^, MEDLINE^®^, MEDLINE^®^ In-Process, the CENTRAL database (structured and systematic reviews), and the Centre for Reviews and Dissemination database (systematic review only) were searched for English-language records from 2009 to 2021 to identify mCRPC treatment guidelines and studies related to the treatment landscape and the humanistic/economic burden of mCRPC in adult men (aged ≥18 years) of any ethnicity.

**Results:**

In total, six records were included for the treatment patterns review, 14 records for humanistic burden, nine records for economic burden, three records (two studies) for efficacy, and eight records for safety. Real-world treatment patterns were broadly aligned with treatment guidelines and provided no optimal treatment sequencing beyond second line other than palliative care. Current post-docetaxel treatments in mCRPC are associated with adverse events that cause relatively high rates of treatment discontinuation or disruption. The humanistic and economic burdens associated with mCRPC are high.

**Conclusion:**

The findings highlight a lack of treatment options with novel mechanisms of action and more tolerable safety profiles that satisfy a risk-to-benefit ratio aligned with patient needs and preferences for patients with late-stage mCRPC. Treatment approaches that improve survival and health-related quality of life are needed, ideally while simultaneously reducing costs and healthcare resource utilization.

## 1 Introduction

Prostate cancer is the most commonly diagnosed cancer among men in developed countries and the second most commonly diagnosed cancer worldwide. Incidence rates are threefold higher in countries with a high human development index score than in those with a medium-to-low score (1–3). Prostate cancer accounted for approximately 6% of all cancer deaths in the United States (US) between 2012 and 2018 (4). Almost three-quarters of cases (73%) were diagnosed with localized (stage I–II) disease; 14% and 7% had regional spread (stage III) and distant metastases (stage IV) at diagnosis, respectively (4). In 2019, 29.2% of patients in England were diagnosed with stage I disease, with 12.9% having stage II, 21.2% having stage III, and 15.7% having stage IV (the stage was reported as “unknown” in 21%) (5). Despite being curable if diagnosed at an early, localized stage, metastatic prostate cancer is still incurable and will inevitably progress to become castration-resistant (6, 7).

Metastatic castration-resistant prostate cancer (mCRPC) is clinically challenging and lethal, with no curative treatment options (8, 9). The 5-year relative survival rate for distant metastatic prostate cancer in the US is currently 32.3% (4). In the United Kingdom (UK), the 5-year survival rate for stage IV prostate cancer is 49%, accounting for 14% of all cancer deaths in men (10). Beyond survival, health-related quality of life (HRQoL) and pain are highly relevant to patients with mCRPC (11). Up to 90% of patients have bone metastases, which are a clinically significant cause of morbidity that often result in severe bone pain, either directly due to metastatic disease or indirectly as a result of symptomatic skeletal events (SSEs) such as fracture and spinal-cord compression (2, 8).

Therapies for mCRPC have evolved greatly over the past decades. In addition to the introduction of androgen receptor pathway inhibitors (ARPIs) in both post-chemotherapy and chemotherapy-naïve settings, asymptomatic and minimally symptomatic patients can undergo treatment with the immunotherapy sipuleucel-T (US only) (12). While ARPIs have improved outcomes in mCRPC, primary or acquired resistance to these agents will ultimately develop in the majority of patients, limiting their effectiveness (7, 13). Docetaxel and cabazitaxel are options for chemotherapy, with a proven survival benefit in the mCRPC setting, and radium (Ra)-223 is a radiopharmaceutical approved for treatment of patients with symptomatic bone metastases and without visceral metastases (12, 14–17).

Recently, the poly (ADP-ribose) polymerase (PARP) inhibitors olaparib and rucaparib were approved as monotherapies in the US, and olaparib also in Europe for patients with mCRPC harboring germline or somatic mutations in DNA damage–repair (DDR) genes (18–21). Olaparib has also been approved by the FDA and EMA in combination with abiraterone for patients with prostate cancer and homologous recombination repair (HHR) mutations, especially BRCA2 (18, 20). In addition, the programmed death receptor 1 inhibitor pembrolizumab is approved in the US for patients with microsatellite instability-high tumors or genetic mutations consistent with Lynch syndrome (21, 22). These advances provide options for select groups of patients, and not all who are treated will benefit (23, 24). Established lines of evidence-based treatments beyond ARPIs and taxane chemotherapy in mCRPC are of limited clinical effectiveness. As such, recommendations in guidelines focus on first-line (1L) and second-line (2L) therapies, with few guidelines existing for treatments in the third-line (3L) setting and beyond (≥3L).

Combinations of ARPIs and PARP inhibitors include olaparib plus abiraterone (PROPEL) (25, 26), enzalutamide plus talazoparib (TALOPRO-2) (27, 28), niraparib plus abiraterone acetate (hereafter “abiraterone”; MAGNITUDE) (29), and enzalutamide with rucaparib (RAMP) (30). All combinations have shown associated benefits of median radiological progression-free survival (rPFS) in patients with mCRPC who harbor HHR mutations – the most frequently altered DDR genes in prostate cancer.

The purpose of this literature review was twofold: to reflect on the current treatment landscape for prostate cancer in the metastatic and castration-resistant setting, and to consider the economic burden of mCRPC. We aimed to identify the unmet needs for patients with late-stage mCRPC and explore the emerging treatment landscape.

## 2 Methods

Three separate structured literature reviews and one systematic literature review (one original and two updates) were conducted to identify publications relating to treatment guidelines, the treatment landscape, and the humanistic and economic burden of mCRPC.

### 2.1 Data sources

#### 2.1.1 Original structured review and systematic literature review

Embase^®^, MEDLINE^®^, MEDLINE^®^ In-Process, and CENTRAL databases were searched for the structured literature reviews; searches took place on June 28, 2019, and included records published between 2009 and 2019 (**Supplemental Tables S1–S3**). Embase^®^, MEDLINE^®^, MEDLINE^®^ In-Process, and the Centre for Reviews and Dissemination database were searched for the systematic literature review for records from 2009 to 2019 using targeted keyword searches for each review; searches took place on September 16, 2019. To supplement the database searches: conference abstracts were searched for the period 2017–2019 from the American Society for Clinical Oncology (ASCO) and European Society for Medical Oncology (ESMO) for all of the reviews; abstracts from the Professional Society for Health Economics and Outcomes Research (ISPOR) and ISPOR Europe conferences were also searched for the economic burden systematic literature review. The National Institute for Health and Care Excellence (NICE) website was searched for the economic burden review. The bibliographies of included systematic reviews/meta-analyses were searched to identify potentially missing studies. Any data gaps were addressed via general internet searches (e.g., Google Scholar).

#### 2.1.2 Updated systematic literature reviews

Updated searches were conducted using MEDLINE^®^, MEDLINE^®^ In-Process, Embase^®^, The Health Technology Assessment (HTA) database, CENTRAL database, the Database of Abstracts of Reviews of Effects (DARE), and the Cochrane Database of Systematic Reviews. EconLit was also searched for the economic and HRQoL reviews. The NICE and Scottish Medicines Consortium (SMC) websites, as well as conference proceedings of ASCO, ESMO, and ISPOR, were searched. Further details are provided in the supplementary methods.

Embase^®^, MEDLINE^®^, MEDLINE^®^ In-Process, and CENTRAL databases were searched for the systematic literature reviews; searches took place on April 6, 2021 (for clinical, economic, and HRQoL records), June 21, 2021 (for HRQoL records), and November 3, 2021 (**Supplemental Tables S4–S9**).

### 2.2 Study selection

The population of interest was adult men (≥18 years of age) of any ethnicity with mCRPC. Only studies published in English were included. Although the economic systematic review was not restricted by geographic location (**Supplemental Table S9**), the following inclusion criteria were applied for the purposes of the present report: studies conducted in France, Germany, Italy, Spain, the UK, or the US in the previous 5 years (2017–2021); 2L therapy and beyond (≥2L) only; and studies with more than 100 participants (if reported) with data on total costs and/or incremental cost-effectiveness ratios (ICERs) or quality-adjusted life years (QALYs) (**Supplemental Tables S4–S9**). For the updated interventional (efficacy) systematic reviews (clinical), only approved/recommended therapies were included in this manuscript and only phase 3 trials were included in the search criteria. Full details of all inclusion/exclusion criteria for all of the searches are provided in the supplemental information.

### 2.3 Review procedure

For the structured literature review, first screening (titles and abstracts) and second screening (full text) were undertaken by a single reviewer followed by a quality check by a second independent reviewer (**Supplemental Figure S1**). Data were extracted by a single reviewer and verified by an independent reviewer. Any discrepancies between the two reviewers for both screening and data extraction were resolved by a third independent reviewer.

For the systematic literature reviews, first screening, second screening, and data extraction were conducted using a two-review process, whereby two independent reviewers performed the screening/data extraction and any discrepancies were resolved by a third reviewer (**Supplemental Figure S1**). The process was conducted in line with the requirements of NICE and in accordance with methodology established in the Preferred Reporting Items for Systematic Reviews and Meta-Analyses (PRISMA) statement (31).

### 2.4 Quality assessment

Quality assessments of the relevant randomized controlled trials and cost-effectiveness and HRQoL studies that were published in full-text were conducted using validated quality assessment tools (32–35).

### 2.5 Attrition

In the initial structured review, a total of 130 records were included in the treatment landscape review, of which 37 reported treatment patterns for mCRPC in a real-world scenario and 25 reported safety outcomes, including two systematic reviews (**Supplemental Figure S2**). The searches and screens for each systematic review are described in **Supplementary Figures S3–S5**.

## 3 Results

The searches and screens for each systematic review detailed in **Supplemental Figure S2** yielded the following:

Interventional (efficacy) systematic literature review (**Supplemental Figure S3**)

1. A total of 9099 records were identified from the database
2. After screening by title/abstract, 1412 records were selected for full-text review
3. A total of 26 records from 20 original studies were selected for data extraction
4. A final total of three records (two original studies) were included in this manuscript

HRQoL systematic literature review (**Supplemental Figure S4**)

1. A total of 1767 records were identified using the Ovid platform
2. After screening by title/abstract, 294 records were selected for full-text review
3. A total of 98 records from 96 original studies were selected for data extraction
4. A final total of 14 records were included in the manuscript

Economic systematic literature review (**Supplemental Figure S5**)

1. A total of 3271 records were identified for economic evaluations from the database search
2. After screening by title/abstract, 456 records were selected for full-text review
3. A total of 74 records from 74 original studies were selected for data extraction
4. A final total of nine records were included in the manuscript

### 3.1 Quality assessment

Based on the quality assessments that were conducted, no papers were excluded from the review. All papers were considered to be of appropriate quality for inclusion.

### 3.2 Treatment landscape

#### 3.2.1 Treatment patterns

Six publications were included (two global, three US, and one Japan). Updated systematic searches were not conducted for treatment patterns (**Table 1**). Considering the limitations in these data, ARPIs, docetaxel, and Ra-223 were commonly prescribed as 1L and 2L treatments, whereas sipuleucel-T was prescribed at 2L only. In the US, abiraterone was the most frequent 1L treatment. At 2L, in Japan, enzalutamide was more frequently prescribed than abiraterone. Among the few patients who received 3L treatment, the most commonly prescribed agents were luteinizing hormone-releasing hormone (LHRH) agonists/antagonists, abiraterone, enzalutamide, docetaxel, and Ra-223. There was limited information about 1L treatment options. Across studies carried out in North America, Latin America, Europe, and Japan, the most commonly used therapies were ARPIs (6.2%–49.6%) (42–44), docetaxel (16.4%–26.9%) (42–44), sipuleucel-T (9.2%–18.6%) (42, 43), and Ra-223 (1.7%–2.7%) (42, 43). Cabazitaxel was used in <2.5% of patients (42–44). In the US, abiraterone was the most prevalent 1L treatment (42.5%–50.5%), followed by docetaxel (16.4%–26.9%), sipuleucel-T (9.2%–18.6%), and enzalutamide (10.3%–18.4%) (42, 43). In Japan, LHRH agonists/antagonists were prescribed in 43.6% of patients in the 1L setting, with antiandrogens, docetaxel, and steroids being given in 26.9%, 19.2%, and 18.7% of cases, respectively (44).

**Table 1:** Studies reporting treatment patterns.

Study name	Country/source	Date	1L		2L		3L	
Global studies								
**Akaza et al., 2018** (36)	Global	November 2011–July 2015			Post-docetaxel: *CT* Any CT 38.3% Taxane 26.4% *Hormonal therapy* Any hormonal therapy 57.5% CYP-17 inhibitors 27.4% Receptor-blocking antiandrogens 11.8% Multi-action antiandrogens 14.6% *Combination* CT and hormonal therapy 13.5%		NR	
			CT was more frequently prescribed in Latin America and in other countries (56.8% and 52.3%, respectively, vs. 27.1% in Europe), whereas a combination of CT and hormonal therapy was less frequently prescribed in Europe (6.6% vs. 20.0%–20.9%). Targeted therapy was more frequently prescribed in Europe (10.7% vs. 0.6%–1.7%)					
**Dizdarevic et al., 2019** (37) **Harshman et al., 2017** (38) **Harshman et al., 2018** (39) **Higano et al., 2017** (40)	Global	September 2014–September 2016	Ra-223 48%		Ra-223 28%		Ra-223 24%	
			Abiraterone or enzalutamide was concomitantly used by 31% of 1L, 29% of 2L, and 14% of ≥3L patients receiving Ra-223. Further, across patients with prior abiraterone or enzalutamide and concomitant abiraterone or enzalutamide, prior docetaxel or cabazitaxel use was reported in 96 (57%) and 46 (30%) patients respectively. Across the 244 patients assessed in the US, use of prior and concomitant abiraterone or enzalutamide was observed in 19% and 34% patients, respectively. In North America and Europe, abiraterone/enzalutamide use was reported in 29% of patients and concomitant in 26%. Across the 564 patients reporting CT subgroups, 190 (34%) received prior CT and 374 (66%) received no prior CT. 123 of 190 and 107 of 374 patients had also received and completed abiraterone or enzalutamide prior to Ra-223. 59 of 190 (31%), and 148 of 374 (40%) patients were treated with concomitant abiraterone and/or enzalutamide.					
North America								
**Oh et al., 2017** (41)	US	NR	100% received docetaxel		Post-docetaxel: Cabazitaxel 19.6% AR-targeted therapy 80.4%		Post cabazitaxel: 56.1% received no further treatment Post AR-targeted therapy: 72.1% received no further treatment	
**Wen et al., 2019** (42)^*^	US	June 2013–September 2014	MarketScan: Abiraterone 49.6% Enzalutamide 18.4% Sipuleucel-T 14.7% Ra-223 2.1% Cabazitaxel 0.8% Docetaxel 16.4% Mitoxantrone 0.1% Antiandrogen 6.2% Other CT 23.4%	PharMetrics: Abiraterone 42.5% Enzalutamide 10.3% Sipuleucel-T 18.6% Ra-223 2.7% Cabazitaxel 1.1% Docetaxel 26.9% Mitoxantrone 0.2% Antiandrogen 7.8% Other CT 25.5%	MarketScan: Abiraterone 37.2% Enzalutamide 20.5% Sipuleucel-T 3.0% Ra-223 1.6% Cabazitaxel 1.3% Docetaxel 14.6% Mitoxantrone 0.2% Antiandrogen 7.8% Other CT 56.6%	PharMetrics: Abiraterone 37.7% Enzalutamide 14.9% Sipuleucel-T 3.0% Ra-223 2.3% Cabazitaxel 1.6% Docetaxel 21.8% Mitoxantrone 0.4% Antiandrogen 8.0% Other CT 62.1%	MarketScan: Abiraterone 33.7% Enzalutamide 21.2% Sipuleucel-T 0.7% Ra-223 2.2% Cabazitaxel 1.5% Docetaxel 12.2% Mitoxantrone 0.2% Antiandrogen 5.8% Other CT 28.3%	PharMetrics: Abiraterone 33.5% Enzalutamide 13.6% Sipuleucel-T 1.2% Ra-223 2.5% Cabazitaxel 1.8% Docetaxel 17.3% Mitoxantrone 0.2% Antiandrogen 6.0% Other CT 31.5%
**Wen et al., 2019** (43)	US	January 2012–June 2015	Abiraterone 50.5% Enzalutamide 15.6% Sipuleucel-T 9.2% Ra-223 1.7% Cabazitaxel 2.3% Docetaxel 22.1%		Abiraterone 35.3% Enzalutamide 22.6% Sipuleucel-T 8.0% Ra-223 1.5% Cabazitaxel 3.2% Docetaxel 18.5%		Abiraterone 21.2% Enzalutamide 16.8% Sipuleucel-T 6.2% Ra-223 2.3% Cabazitaxel 5.0% Docetaxel 13.2%	
Asia								
**Uemura et al., 2017** (44)^*^	Japan	December 2014–February 2015	LHRH 43.64% Antiandrogens 26.93% Docetaxel 19.20% Steroid 18.70% Enzalutamide 14.46% Bisphosphonate 13.72% Denosumab 10.72% Estramustine 10.22% NSAIDs 8.98% Abiraterone 8.73% CAB 7.73% Opioid 4.49% Strontium-89 1.25% EBRT 0.75% Cabazitaxel 0.50% Ketoconazole 0.25%		LHRH 46.62% Antiandrogens 24.20% Docetaxel 13.17% Steroid 23.13% Enzalutamide 25.98% Bisphosphonate 17.79% Denosumab 13.52% Estramustine 11.03% NSAIDs 11.74% Abiraterone 9.96% CAB 6.76% Opioid 6.41% Strontium-89 1.07% EBRT 2.14% Cabazitaxel 1.07% Ketoconazole 0.00%		LHRH 41.22% Antiandrogens 8.11% Docetaxel 26.35% Steroid 35.14% Enzalutamide 30.41% Bisphosphonate 21.62% Denosumab 15.54% Estramustine 8.11% NSAIDs 9.46% Abiraterone 18.92% CAB 4.73% Opioid 8.11% Strontium-89 0.68% EBRT 2.7% Cabazitaxel 1.35% Ketoconazole 0.00%	

^*^Fourth-line setting also reported but not included here.

1L, first line; 2L, second line; 3L, third line; AR, androgen receptor; CAB, combined androgen blockade; CT, chemotherapy; CYP-17, cytochrome P450 17A1; EBRT, external beam radiotherapy; LHRH, luteinizing hormone-releasing hormone agonist/antagonist; mCRPC, metastatic castration-resistant prostate cancer; NA, not applicable; NR, not reported; NSAIDs, nonsteroidal anti-inflammatory drugs; Ra-223, radium-223; US, United States.

The most common 2L treatments were ARPIs (7.8%–37.7%), followed by docetaxel (13.2%–21.8%) and Ra-223 (1.5%–2.3%) (40, 42–44). Use of cabazitaxel was limited in the 2L setting (<3.5%) (42–44). In the US, ARPIs were the predominant 2L therapy, the most frequently prescribed being abiraterone (35.3%–37.7%) followed by enzalutamide (14.9%–22.6%) (42, 43). In one US study that included only patients who had received 1L docetaxel, 80.4% received an ARPI as a 2L treatment and 19.6% received cabazitaxel (41). Undertreatment in the ≥3L setting was reported in 56.1%–72.1% of patients in a US cohort (41). One global study noted that chemotherapy was more frequently prescribed as a 2L option in Latin America and in other countries, including Algeria, Iran, Pakistan, Saudi Arabia, Turkey, and United Arab Emirates (56.8% and 52.3%, respectively), compared with Europe (27.1%), as was combination chemotherapy and hormonal therapy (20.0%–20.9% vs. 6.6%); however, targeted therapy was more frequently prescribed in Europe (10.7% vs. 0.6%–1.7% in other countries) (36). In Japan, enzalutamide was a more popular choice (26.0%) than abiraterone (10.0%), and 24.2% of patients received antiandrogens, although the most common 2L therapy was LHRH (46.6%) (44). There appears to be no consensus regarding treatment sequencing.

A large proportion of patients do not receive treatment beyond 2L (69%–91%) (36, 41), which may explain the dearth of studies in this setting. Among those who receive 3L treatment, the most commonly prescribed agents are LHRH agonists/antagonists (41.2%), abiraterone (18.9%–33.7%), enzalutamide (13.6%–30.4%), docetaxel (12.2%–26.4%), and Ra-223 (2.2%–2.5%) (42, 43). A global study reported that the most frequent 3L treatment was hormonal therapies (50.6%), chemotherapy (32.1%), and palliative radiotherapy (18.5%) (36). Only one study, out of Japan, reported the use of LHRH agonists/antagonists, most frequently prescribed at 3L, followed by enzalutamide (30.4%) and docetaxel (26.4%) (44). In the US, abiraterone is the most common 3L treatment (21.2%–33.7%), followed by enzalutamide (13.6%–21.2%) and docetaxel (12.2%–17.3%); rates for all other named treatment options were <7% (42, 43). As with 1L and 2L, use of cabazitaxel is limited in the 3L setting globally.

#### 3.2.2 Efficacy

Most pivotal phase 3 trials for currently approved and prescribed treatments (e.g., prostate-specific membrane antigen radioligand therapy [^177^Lu-PSMA-617], rucaparib, Ra-223, pembrolizumab) were published outside the scope of this systematic review (which only included papers published between 2017 and 2021). The clinical trials included in this review are the PROfound trial of olaparib versus enzalutamide plus abiraterone (two papers) and the CARD trial of cabazitaxel versus enzalutamide or abiraterone plus prednisone (**Table 2**).

**Table 2:** Summary of efficacy of approved treatments for mCRPC (only studies identified in systematic review*).

Study name	N	Patient population	Inter-vention	Efficacy				
				OS (mo; 95% CI)	OS HR (95% CI)	PFS (mo; 95% CI)	PFS HR (95% CI)	ORR (%)
PROfound NCT02987543, global phase 3 trial^†,‡^ (45, 46)	256	Patients with mCRPC who have progressed on prior hormonal agent Alteration in any of the 15 prespecified genes (Cohorts A and B)	Olaparib	17.3	0.79 (0.61–1.03) *P* value NR	rPFS: 5.8	0.49 (0.38–0.63) *P <* 0.001	22
	131		Enzalutamide or abiraterone	14.0		rPFS: 3.5		4
PROfound NCT02987543, global phase 3 trial^†,‡^ (45, 46)	162	Patients with mCRPC who have progressed on prior hormonal agent: Cohort A	Olaparib	19.1	0.69 (0.5–0.97) *P =* 0.02	rPFS: 7.4	0.34 (0.25–0.47) *P <* 0.001	33
	83		Enzalutamide or abiraterone	14.7		rPFS: 3.6		2
PROfound NCT02987543, global phase 3 trial^†,‡^ (45, 46)	94	Patients with mCRPC who have progressed on prior hormonal agent: Cohort B	Olaparib	14.1	0.96 (0.63–1.49) *P* = NR	4.8	0.88 (0.58–1.36)	NR
	48		Enzalutamide or abiraterone	11.5		3.3		NR
CARD NCT02485691 Global phase 3 trial (47)	129	Patients with progressive mCRPC who had been treated with three or more cycles of docetaxel	Cabazitaxel	13.6 (11.5–17.5)	0.64 (0.64–0.89) *P =* 0.008	rPFS: 8.0 (5.7–9.2)	rPFS HR: 0.54 (0.40–0.73) *P <* 0.001	36.5
						PFS: 4.4 (3.6–5.4)	PFS HR: 0.52 (0.40–0.68) *P =* 0.001	
	126		Enzalutamide or abiraterone	11.0 (9.2–12.9)		rPFS: 3.7 (2.8–5.1)		11.5
						PFS: 2.7 (2.4–2.8)	PFS HR: 0.52 (0.40–0.68) *P <* 0.001	

*Only two studies (PROfound and CARD) were identified in the systematic review. For completeness, additional pivotal clinical trials are included in **Supplemental Table S15**.

^†^Final OS reported from Hussain et al., 2020 (45).

^‡^Final PFS reported from de Bono et al., 2020 (46).

CI, confidence interval; HR, hazard ratio; mCRPC, metastatic castration-resistant prostate cancer; mo, months NR, not reported; ORR, objective response rate; OS, overall survival; PFS, progression-free survival; rPFS, radiographic progression-free survival.

Median overall survival (OS) of patients treated with abiraterone or enzalutamide ranged from 11.0 to 14.7 months (45–47). Median radiographic progression free survival (rPFS) or progression-free survival (PFS) of patients treated with abiraterone or enzalutamide ranged from 2.7 to 3.7 months (45–47). Median OS of patients treated with olaparib was 14.1–19.1 months, and median rPFS was 5.8–7.4 months (45, 46). Patients treated with cabazitaxel had median OS and rPFS durations of 13.6 and 8.0 months, respectively (47). The patient populations differed between these two trials: the PROfound study enrolled patients with mCRPC who had qualifying alterations in homologous recombination repair (HRR) genes and who had progressed after treatment with an ARPI (45), whereas patients in the CARD trial had been previously treated with docetaxel and an ARPI (47).

#### 3.2.3 Safety

This report includes six real-world evidence studies from the structured review and three papers reporting two original clinical trials from the interventional systematic review (**Table 3**; **Supplemental Table S14**). Safety evidence from these studies is summarized below; however, due to the heterogeneity between study designs and patient populations, data must be interpreted with caution.

**Table 3:** Summary of included studies assessing safety of current approved/recommended mCRPC treatments.

Author	Population	Intervention	Country	Sample size	Results
Real-world evidence studies					
Beardo et al., 2019 (48)	Elderly CT-naïve (patients had failed ADT)	Abiraterone or enzalutamide	Spain	134	• Incidence of AEs 46.3% • Grade 3 and 4 AEs 14.2% • Asthenia of any type was more common with administration of enzalutamide than abiraterone (15% vs. 4.3%, *P =* 0.04) • Dose reduction 6.7% (10% in patients treated with enzalutamide vs. 5.3% abiraterone; *P =* 0.261) • Temporary treatment discontinuation due to AEs in 12.7% of patients, with no significant differences between age groups. Treatment discontinuation was more frequent with enzalutamide (25% vs. 11.9% with AA; *P =* 0.012)
Carles et al., 2019 (49)	Previously treated with docetaxel	Cabazitaxel	Lebanon, Czech Republic, Spain, Austria, Russia, and Bulgaria	189	• Any grade TEAEs were reported in 37.6% of patients, and grade 3/4 TEAEs were reported in 13.8% of patients • Clinical neutropenia (7.9%) and anemia (2.1%) were the most common grade ≥3 TEAEs • TEAEs possibly related to cabazitaxel were anemia (10.6%), neutropenia (9.5%), diarrhea (8.5%), and asthenia (7.9%). Clinical neutropenia (7.9%) and anemia (2.1%) were the most common grade ≥3 TEAEs • Serious TEAEs possibly related to cabazitaxel were reported in 12.2% of patients, the most common of which was neutropenia (5.8%)
Gonzalez del Alba et al., 2017 (50)	After 1L docetaxel	Abiraterone, cabazitaxel	Spain	150	Most common toxicities of special interest (all grades/grade 3–4 as % of patients): • Fatigue (31/1% for abiraterone vs. 54/4% for cabazitaxel) • Edema (15/0% for abiraterone vs. 13/4% for cabazitaxel) • Hypertension (7/1% for abiraterone) • Diarrhea (8/0% for abiraterone vs. 31/4% for cabazitaxel) • Vomiting (9/2% for abiraterone vs. 11/0% for cabazitaxel) • Neutropenia (7/4% for cabazitaxel)
Dizdarevic et al., 2019 (37)	Prior and no prior CT, results given	Ra-223	REASSURE, Global	583	• Any AEs were experienced by 300 of 564 evaluable patients: 63% in the prior CT group and 48% in the CT naïve group • Drug-related TEAEs were reported in 213 patients (38%), 41% in the prior CT group and 36% in the CT-naïve group • Gastrointestinal disorders (21% [22%, 21%]) and hematological toxicities (13% [21%, 9%]) were the most common TEAEs • Anemia was the most common grade 3/4 TEAE (4% [8%, 2%]) • Four drug-related deaths were reported, all in the prior chemotherapy group • Treatment discontinuations due to drug-related TEAEs were observed in 6% [CT-treated: 9%, CT-naïve: 5%] of patients • Drug-related SAEs occurred in 4% (CT-treated: 7%, CT-naïve: 3%) of patients, the most common were hematological toxicities reported in 13 patients (eight and five patients, respectively)
Fourrier-Reglat et al., 2018 (51)	2L setting post-docetaxel	Cabazitaxel	France	401	• Grade 3 AEs were reported in 55.4% of patients and were mainly hematological (39.9%; anemia: 26.9%, neutropenia: 15%, febrile neutropenia: 8%) and urinary (9.2%; renal failure: 7.2%) • Septicemia/septic shock was reported in 5% of patients, and of these, six patients died due to septicemia
Sirotova et al., 2018 (52)	Pre- and post-CT in elderly (≥75 years)	Abiraterone	Italy	252	• 3.2% discontinued due to toxicity • 1.6% temporary dose reductions • Most frequent grade 3 toxicities were hypertension (1.6%) and liver toxicity (2%)
Parente et al., 2017 (53)	Previously treated with docetaxel	Cabazitaxel	Australia	104	• Grade ≥3 TEAEs of clinical concern and grade ≥4 TEAEs were experienced by 64.4% and 29.8% of patients, respectively • Neutropenia, febrile neutropenia, diarrhea, and vomiting were the most frequent grade ≥3 TEAEs • Six patients experienced a TEAE leading to death. 21.2% of patients experienced dose delays due to cabazitaxel toxicity
Summers et al., 2017 (54)	Post-docetaxel therapies	Post-docetaxel therapies	NR	NR	• Pooled European data from the cabazitaxel EAP (1301 participants) listed an incidence of any AE ranging from 83.4% to 88.3%, and incidence of grade ≥3 AEs ranging from 47% to 56.6% The final analysis of abiraterone EAP reported grade ≥3 AEs in 41% of 2314 patients • By contrast, a lower incidence of grade ≥3 AEs (14.2%) was reported in the largest study from the enzalutamide EAP
Phase 3 RCTs					
De Witt et al., 2019 (47)	Patients with progressive mCRPC who had been treated with three or more cycles of docetaxel and an ARPI	Cabazitaxel	Austria, Belgium, Czech Republic, France, Germany, Greece, Iceland, Ireland, Italy, Netherlands, Norway, Spain, UK	126	• Grade ≥3 AEs: 56% • SAEs: 39% • AEs leading to treatment discontinuation: 19.8% • AEs leading to death: 5.6% • Most common AEs were asthenia or fatigue (53%), diarrhea (40%), infection (32%), and musculoskeletal pain or discomfort (27%)
		Enzalutamide or abiraterone		124	• Grade ≥3 AEs: 52% • SAEs: 39% • AEs leading to treatment discontinuation: 8.9% • AEs leading to death: 11.3% • Most common AEs were musculoskeletal pain or discomfort (40%), asthenia or fatigue (37%), nausea or vomiting (23%), and infection (20%)
Hussain et al., 2020 (45)	Patients with mCRPC who have progressed on prior hormonal agents – Cohorts A and B	Olaparib	Argentina, Australia, Austria, Brazil, Canada, Denmark, France, Germany, Israel, Italy, Japan, Netherlands, Norway, South Korea, Spain, Sweden, Taiwan, Turkey, UK, US	256	• Grade ≥3 AEs: 52% • SAEs: 30% • Most common related AEs were anemia (39%), nausea (36%), and fatigue or asthenia (32%) • Olaparib was discontinued because of anemia in 7% of patients and because of neutropenia, thrombocytopenia, nausea, vomiting, or fatigue or asthenia in 1% of patients for each • AEs led to death in 4% of patients in both the olaparib and crossover treatment groups. One death was treatment-related, due to pneumonia and neutropenia
		Enzalutamide or abiraterone		131	• Grade ≥3 AEs: 40% • SAEs: 37% • Most common related AEs were fatigue or asthenia (21%), nausea (11%), and decreased appetite (7%) • Treatment-related fatigue or asthenia led to treatment discontinuation in 2% of patients • AEs led to death in 5% of patients: one death was treatment-related due to pleural effusion

1L, first line; 2L, second line; ADT, androgen deprivation therapy; AE, adverse event; ARPI, androgen receptor pathway inhibitor; CT, chemotherapy; EAP, expanded access program; mCRPC, metastatic castration-resistant prostate cancer, NR, not reported; Ra-223, radium-223; RCT, randomized controlled trial; SAE, serious adverse event; TEAE, treatment-emergent adverse event; UK, United Kingdom; US, United States.

##### 3.2.3.1 Cabazitaxel

The most commonly reported grade ≥3 treatment-emergent adverse events (TEAEs) experienced by patients treated with cabazitaxel (all previously treated with docetaxel) were hematological, comprising neutropenia (including febrile neutropenia), anemia, leukopenia, and thrombocytopenia. Diarrhea, vomiting, asthenia, renal failure, and septicemia/septic shock were also reported in real-world settings (49–51, 53). In a clinical trial in patients who had been pretreated with docetaxel and had progressed on an ARPI (47), additional common TEAEs of infection and musculoskeletal pain or discomfort were reported (**Table 3**) (49, 51, 53).

##### 3.2.3.2 ARPI

The most common toxicities of special interest in a Spanish real-world setting were fatigue, edema, hypertension, diarrhea, and vomiting (50). In elderly Italian patients treated with abiraterone, a grade 3 TEAE of liver toxicity was also reported in 2% of patients (**Table 3**) (52). In clinical trials of treatment with enzalutamide or abiraterone, the most commonly reported TEAEs were musculoskeletal pain or discomfort, fatigue or asthenia, infection, nausea, vomiting, and decreased appetite (**Table 3**) (45, 47). As expected, in the CARD trial there were no reports of febrile neutropenia with 2L ARPI treatment, compared with 3.2% of patients receiving 2L cabazitaxel (47).

##### 3.2.3.3 Ra-223

Hematological and gastrointestinal TEAEs were the most frequently reported toxicities in patients who had received prior chemotherapy. Anemia was the most common grade 3/4 TEAE, and was four times more common in patients who had received prior chemotherapy compared with those who had not (8% vs. 2%) (37). In addition, grade 3/4 thrombocytopenia occurred at double the rate in patients who had previously received chemotherapy versus those who had not (4% vs. 2%).

##### 3.2.3.4 Olaparib

Olaparib was associated with more grade ≥3 TEAEs and fewer serious adverse events (SAEs) than enzalutamide or abiraterone (**Table 3**). In the global randomized phase 3 PROfound trial of olaparib in patients with mCRPC who had progressed on an ARPI, the most common TEAEs were anemia, nausea, and fatigue or asthenia (45).

### 3.3 Humanistic burden

Studies reporting the impact of intervention included real-world, observational, prospective, retrospective, and cross-sectional studies, as well as surveys and randomized controlled trials. Across the included studies, HRQoL was assessed using multiple patient-reported outcome (PRO) measures. The application of additional inclusion criteria for this manuscript, limiting inclusion to full papers published in the last 5 years and with a study population of more than 100 receiving ≥2L therapy, yielded 14 papers reporting HRQoL endpoints (**Table 4**; **Supplemental Figure 2**), including the EuroQol five-dimension questionnaire (EQ-5D; five papers), Functional Assessment of Cancer Therapy-General/Prostate questionnaire (FACT-G/P; 11 papers), Brief Pain Inventory-Short Form questionnaire (BPI-SF; four papers), Short Form-36 questionnaire (SF-36; one paper), Brief Fatigue Inventory (BFI; two papers), Australian Quality of Life-8 dimension questionnaire (AQoL-8D; one paper), and European Organization for Research and Treatment of Cancer Quality of Life Questionnaire (EORTC QLQ; one paper) (**Table 4**).

**Table 4:** Summary of HRQoL in included studies with patients with prostate cancer.

Measure	Author	Study characteristics	HRQoL summary
**EQ-5D**	Carles et al., 2019 (49)	Prospective, observational study (CAPRISTANA) in patients with mCRPC previously treated with docetaxel (2L+/N=189). Patients were treated with cabazitaxel	EQ-5D VAS • Change in VAS score at cycle 2, mean ± SD: 2.7 ± 16.9 • Change in VAS score at cycle 3, mean ± SD: 3.8 ± 10.5 • Change in VAS score at cycle 4, mean ± SD: 7.3 ± 17.8 • Change in VAS score at cycle 5, mean ± SD: 6.2 ± 18.1 • Change in VAS score at cycle 6, mean ± SD: 8.5 ± 25.3 • Change in VAS score at cycle 7, mean ± SD: –2.7 ± 20.5 • Change in VAS score at cycle 8, mean ± SD: 8.4 ± 22.3 • Change in VAS score at cycle 9, mean ± SD: 4.7 ± 20.1 • Change in VAS score at cycle 10, mean ± SD: 7.3 ± 14.9
	Dearden et al., 2019 (55)	Survey in patients with mCRPC who had been taking abiraterone or enzalutamide treatment for ≥2 months (2L+/N=152)	EQ-5D VAS* • Abiraterone arm, pre-chemotherapy score: 72.8 • Enzalutamide arm, pre-chemotherapy score: 68.6 • Abiraterone arm, post-chemotherapy score: 66.3 • Enzalutamide arm, post-chemotherapy score: 67.2
	Fizazi et al., 2020 (56)	Randomized controlled trial (CARD) in patients with mCRPC previously treated with ≥3 cycles docetaxel (2L+/N=255)	EQ-5D VAS • LS mean difference between cabazitaxel and abiraterone or enzalutamide ranging from 1.6 (95% CI –3.65 to 6.85) to 6.4 (1.49 to 11.25; *P =* 0.060)
	Murasawa et al., 2019 (57)	Cross-sectional observational study of men with PC (N=380, including n=38 with mCRPC)	EQ-5D-5L (total cohort vs. mCRPC) • Mean ± SD = 0.86 ± 0.16; 0.84 ± 0.17EQ-5D VAS* • Mean ± SD = 74.6 ± 16.8; 66.3 ± 20.6
	Payne et al., 2022 (58)	European, prospective, observational study of enzalutamide in patients with mCRPC: PREMISE	EQ-5D VAS* Cohort 1 – chemotherapy-naïve plus abiraterone-naïve (n=1171), n (%) • 3 months: Improve: 238 (27.2); No change: 410 (46.9); Worsen: 227 (25.9) • 6 months: Improve: 184 (27.6); No change: 292 (43.9); Worsen: 189 (28.3) • 9 months: Improve: 160 (30.0); No change: 213 (40.1); Worsen: 158 (29.7) Cohort 2 – postchemotherapy plus abiraterone-naïve (n=438), n (%) • 3 months: Improve: 117 (34.3); No change: 116 (34.0); Worsen: 108 (31.7) • 6 months: Improve: 87 (39.0); No change: 83 (37.2); Worsen: 53 (23.7) • 9 months: Improve: 53 (32.9); No change: 65 (40.4); Worsen: 43 (26.7)
**EORTC QLQ-INFO25**	Jenkins et al., 2019 (59)	Mixed-methods observational study in men with mCRPC (N=132)	EORTC QLQ-INFO25 • Mean ± SD global score at baseline = 58.49 ± 18.57; mean change at 6 months –1.01 (95% CI –3.57 to 1.57, *P =* 0.44)
**FACT**	Badrising et al., 2021 (60)	Non-interventional, multicenter, prospective, observational registry study of patients with mCRPC scheduled to be treated with Ra-223	Time to FACT-P deterioration (months) Overall cohort (n=105) Total FACT-P • Median (IQR): 5.7 (3.3–NR) • Mean (95% CI): 7.8 (6.2–9.3) Patients with pain at baseline (n=45) • Median (IQR): 13.7 (2.5–NR) • Mean (95% CI): 8.4 (6.4– 10.5) Patients without pain at baseline (n=60) • Median (IQR): 5.5 (3.1–NR) • Mean (95% CI): 7 (5.4–8.6) Clinically meaningful improvement in Total FACT-P during treatment, n (%)^†^ • Overall cohort: 33 (31.4) • Patients with pain at baseline: 17 (37.7) • Patients without pain at baseline: 16 (26.7)
	Carles et al., 2019 (49)	Prospective, observational study (CAPRISTANA) in patients with mCRPC previously treated with docetaxel (2L+/N=189)	• FACT-P score at baseline, median (Q1–Q3): 96.0 (83.0–114.5) • FACT-P score improved over time, n (%): 48 (32.2) • FACT-P score stable over time, n (%): 60 (40.3) • FACT-P score deteriorated over time, n (%): 41 (27.5)
	Fizazi et al., 2020 (56)	Randomized controlled trial (CARD) in patients with mCRPC previously treated with ≥3 cycles docetaxel (2L+/N=255)	FACT-P mean change from baseline: • Cabazitaxel arm: –6.33 (SE: 2.81) • Abiraterone or enzalutamide arm: –10.91 (SE 3.13) ◦ LS mean difference 4.58, 95% CI −1.36 to 10.52, *P =* 0.13 Time to deterioration in months, median (95% CI): • Cabazitaxel: 14.8 (6.3–NE) • Abiraterone or enzalutamide arm: 8.9 (6.3–NE)
	Guo et al., 2019 (61)	Prospective, observational study in patients with mCRPC previously treated by androgen deprivation therapy (2L+/N=134)	FACT-P • Enzalutamide arm at baseline (mean ± SD): 118.4 ± 20.6, *P =* 0.112 • Bicalutamide arm at baseline (mean ± SD): 112.8 ± 19.3, *P =* 0.112 FACT-G • Enzalutamide arm at baseline (mean ± SD): 85.1 ± 14.9, *P =* 0.350 • Bicalutamide arm at baseline (mean ± SD): 82.7 ± 14.2, *P =* 0.350 FACT-G – Enzalutamide better improved HRQoL assessed by FACT-G and FACT-P compared with bicalutamide in mCRPC patients
	Jenkins et al., 2019 (59)	Mixed-methods observational study in men with mCRPC (N=132)	FACT-P • Significant decline at 6 months: mean = –3.89, 95% CI: 6.7–1.05, *P =* 0.007 • Quality of life declined (3 months), n (%): 41 (35%) • Quality of life no change (3 months), n (%): 45 (38%) • Quality of life improved (3 months), n (%): 32 (27%) • Quality of life declined (6 months), n (%): 48 (45%) • Quality of life no change (6 months), n (%): 33 (31%) • Quality of life improved (6 months), n (%): 25 (24%) FACT-G • Mean = –3.45, 95% CI: –5.53 to –1.36, *P =* 0.002 • Quality of life declined (3 months), n (%): 10 (34%) • Quality of life no change (3 months), n (%): 49 (42%) • Quality of life improved (3 months), n (%): 29 (25%) • Quality of life declined (6 months), n (%): 42 (40%) • Quality of life no change (6 months), n (%): 42 (41%) • Quality of life improved (6 months), n (%): 21 (20%)
	McKay et al., 2017 (62)	Real-world study of men with CRPC and bone metastases (N=832)	FACT-P • Mean ± SD functional well-being score for patients with mCRPC with SSE = 17.5 ± 7.1 vs. non-SSE cohort = 19.8 ± 6.1; *P =* 0.158, with an effect size of 0.36 indicating a meaningful difference
	Murasawa et al., 2019 (57)	Cross-sectional observational study of men with PC (n=380 including N=38 with mCRPC)	FACT-P (Total cohort vs. mCRPC) • Total score: mean ± SD = 110.8 ± 19.6 vs. 105.0 ± 16.4 • Physical well-being: mean ± SD = 24.7 ± 3.9 vs. 22.5 ± 4.3 • Social well-being: mean ± SD = 14.5 ± 7.4 vs. 15.7 ± 5.2 • Emotional well-being: mean ± SD = 19.3 ± 4.0 vs. 17.0 ± 3.8 • Functional well-being: mean ± SD = 19.0 ± 7.1 vs. 18.3 ± 5.4 • PC subscale: mean ± SD = 33.3 ± 7.0 vs. 31.4 ± 5.5
	Payne et al., 2022 (58)	European, prospective, observational study of enzalutamide in patients with mCRPC: PREMISE	FACT-P Total Score Cohort 1 – chemotherapy-naïve plus abiraterone-naïve (n=1171) • 3 months: Improve: 267 (31.3); No change: 319 (37.4); Worsen: 266 (31.2) • 6 months: Improve: 206 (31.5); No change: 207 (31.7); Worsen: 241 (36.9) • 9 months: Improve: 178 (34.4); No change: 163 (31.5); Worsen: 176 (34.0) Cohort 2 – postchemotherapy plus abiraterone-naïve (n=438) • 3 months: Improve: 112 (33.1); No change: 91 (26.9); Worsen: 135 (39.9) • 6 months: Improve: 73 (33.8); No change: 70 (32.4); Worsen: 73 (33.8) • 9 months: Improve: 47 (29.9); No change: 48 (30.6); Worsen: 62 (39.5)
	Saad et al., 2017 (63)	Patients with mCRPC experiencing at least one SRE during AFFIRM (mCRPC previously treated with one or two chemotherapy regimens) and PREVAIL (patients with asymptomatic or mildly symptomatic chemotherapy-naïve mCRPC, despite androgen deprivation therapy) trials	PREVAIL trial FACT-P Total Score, TAMC (95% CI) • Patients with any SSE (n=156): –5.11 (–7.85 to –2.37)^¶^ • Patients with radiation or surgery to bone (n=103): –2.00 (–4.86 to 0.85) • Patients with a pathologic bone fracture (n=29): –4.07 (–10.93 to 2.80) • Patients with a spinal cord compression (n=23): –16.95 (–26.47 to –7.44)^¶^ FACT-P PCS, TAMC (95% CI) • Patients with any SSE (n=161): –1.92 (–2.91 to –0.94)^¶^ • Patients with radiation or surgery to bone (n=106): –0.72 (–1.77 to 0.33) • Patients with a pathologic bone fracture (n=30): –2.45 (–5.04 to 0.13) • Patients with a spinal cord compression (n=23): –6.00 (–9.30 to –2.69)^¶^ FACT-G Total Score, TAMC (95% CI) • Patients with any SSE (n=158): –3.41 (–5.30 to –1.52)^¶^ • Patients with radiation or surgery to bone (n=105): –1.85 (–3.92 to 0.23) • Patients with a pathologic bone fracture (n=29): –1.49 (–5.99 to 3.00) • Patients with a spinal cord compression (n=23): –10.72 (–17.14 to –4.29)^¶^ AFFIRM trial FACT-P Total Score, TAMC (95% CI) • Patients with any SSE (n=139): –6.94 (–9.93 to –3.95)^¶^ • Patients with radiation or surgery to bone (n=96): –6.69 (–10.26 to –3.12)^¶^ • Patients with a pathologic bone fracture (n=23): –7.62 (–16.80 to 1.56) • Patients with a spinal cord compression (n=26): –9.69 (–16.10 to –3.27)^¶^ FACT-P PCS, TAMC (95% CI) • Patients with any SSE (n=139): –1.79 (–2.91 to –0.68)^¶^ • Patients with radiation or surgery to bone (n=96): –1.37 (–2.60 to –0.13)^¶^ • Patients with a pathologic bone fracture (n=23): –2.80 (–6.82 to 1.22) • Patients with a spinal cord compression (n=26): –2.66 (–5.01 to –0.32)^¶^ FACT-G Total Score, TAMC (95% CI) • Patients with any SSE (n=142): –5.46 (–7.55 to –3.36)^¶^ • Patients with radiation or surgery to bone (n=99): –5.57 (–8.13 to –3.01)^¶^ • Patients with a pathologic bone fracture (n=23): –5.18 (–10.88 to 0.51) • Patients with a spinal cord compression (n=26): –6.96 (–11.97 to –1.94)^¶^
	Stenner et al., 2017 (64)	Swiss multicenter registry was a prospective, longitudinal, noninterventional study in patients with mCRPC which had progressed after 1L treatment with docetaxel (2L/N=138)	FACT-P Total Score, mean ± SD Overall cohort (n=138) • Visit 1 (n=138): 104.7 ± 19.6 • Visit 2 (n=122): 107.1 ± 21.9 • Visit 5 (n=93): 109.9 ± 20.9 • Visit 7 (n=85): 107.0 ± 22.3
	Thiery-Vuillemin et al., 2021 (65)	Retrospective study of PROSELICA clinical trial in patients with mCRPC that previously received docetaxel (2L+; N=1200). Patients with mCRPC receiving cabazitaxel in the phase III PROSELICA (post-docetaxel) trial and docetaxel + cabazitaxel in FIRSTANA (chemotherapy naïve) trial, respectively	PROSELICA trial FACT-P Total Score improvements • C20: 38.8% • C25: 40.5% FACT-P Subscale score improvements Physical well-being score • C20: 26.4% • C25: 32.1% Social well-being score • C20: 26.4% • C25: 32.1% Emotional well-being score • C20: 30.5% • C25: 31.8% Functional well-being score • C20: 27.7% • C25: 24.6% Prostate-specific concerns score • C20: 42.0% • C25: 49.4% FIRSTANA: FACT-P Total Score improvements • C20: 43.4% • C25: 49.7% • D75: 44.9% FACT-P Subscale score improvements Physical well-being score • C20: 29.5% • C25: 28.0% • D75: 25.6% Social well-being score • C20: 21.0% • C25: 20.8% • D75: 16.6% Emotional well-being score • C20: 43.2% • C25: 38.8% • D75: 37.0% Functional well-being score • C20: 33.1% • C25: 26.6% • D75: 28.7% Prostate-specific concerns score • C20: 52.7% • C25: 55.1% • D75: 54.6%
**BPI-SF**	Badrising et al., 2022 (60)	Non-interventional, multicenter, prospective, observational registry study of patients with mCRPC scheduled to be treated with Ra-223	Time to BPI-SF deterioration (months) Overall cohort (n=105) Worst pain/time to pain progression • Median (IQR): 5.6 (4.7–9) • Mean (95% CI): 7.9 (6.4–9.4) Patients with pain at baseline (n=45) • Median (IQR): 11.1 (7.6–NR) • Mean (95% CI): 8.5 (6.4–13.8) Patients without pain at baseline (n=60) • Median (IQR): 4.1 (3.6–5.7) • Mean (95% CI): 6.1 (4.6–7.7) Clinically meaningful improvement in BPI-SF worst pain during treatment, n (%)^‡^ • Overall cohort: 52 (59.5) • Patients with pain at baseline: 35 (77.7) • Patients without pain at baseline: 17 (28.3) *P =* 0.001 pain at baseline vs. no pain at baseline for worst pain/time to progression. *P* < 0.0001 pain at baseline vs. no pain at baseline for clinically meaningful improvement
	Jenkins et al., 2019 (59)	Mixed-methods observational study in men with mCRPC (N=132)	Clinically meaningful changes in pain intensity (BPI-SF Q3) • Proportion of patients with no or little pain at baseline (n=69) experiencing improvement at 3 months = 6% (4/63) and at 6 months = 5% (3/58) • Proportion of patients with moderate/severe pain at baseline (n=57) experiencing improvement at 3 months = 43% (20/47) and at 6 months = 40% (16/40)
	McKay et al., 2017 (62)	Real-world study of men with CRPC and bone metastases (N=832)	• Pain severity score (all patients): mean ± SD = 1.8 ± 2.0^§^ • Mean pain severity score was significantly higher in the SSE vs. non-SSE cohort (2.5 ± 2.2 vs. 1.6 ± 1.9; *P =* 0.048; ES=0.47)
	Payne et al., 2022 (58)	European, prospective, observational study of enzalutamide in patients with mCRPC: PREMISE	BPI-SF severity, n (%) Cohort 1 – chemotherapy-naïve plus abiraterone-naïve (n=1171) • 3 months; Improve: 82 (10.0); No change: 640 (77.8); Worsen 101 (12.3) • 6 months; Improve: 71 (11.4); No change: 466 (74.6); Worsen 88 (14.1) • 9 months: Improve: 55 (10.9); No change: 378 (75.2); Worsen 70 (13.9) Cohort 2 – postchemotherapy plus abiraterone-naïve (n=438) • 3 months: Improve: 53 (16.3); No change: 237 (72.2); Worsen: 36 (11.0) • 6 months: Improve: 33 (15.2); No change: 160 (73.7); Worsen 24 (11.1) • 9 months: Improve: 19 (13.4); No change: 108 (76.1); Worsen: 15 (10.6) BPI-SF interference, n (%) Cohort 1 – chemotherapy-naïve plus abiraterone-naïve (n=1171) • 3 months: Improve: 166 (20.5); No change: 475 (58.6); Worsen: 169 (20.9) • 6 months: Improve: 135 (22.3); No change: 364 (60.2); Worsen: 106 (17.5) • 9 months: Improve: 102 (21.1); No change: 284 (58.7); Worsen: 98 (20.3) Cohort 2 – postchemotherapy plus abiraterone-naïve (n=438) • 3 months: Improve: 76 (23.9); No change: 171 (53.8); Worsen: 71 (22.3) • 6 months: Improve: 50 (23.6); No change: 110 (51.9); Worsen: 52 (24.5) • 9 months: Improve: 32 (22.9); No change: 78 (55.7); Worsen: 30 (21.4)
**SF-36**	Shultz et al., 2019 (66)	Survey of patients with mCRPC enrolled in Medicare Advantage or Prescription Drug plan treated with enzalutamide or abiraterone (2L+/N=269)	• SF-36-PCS, mean ± SD: 39.9 ± 10.6 • SF-36-MCS, mean ± SD: 53.4 ± 10.4 Physical health was impacted more than mental health in these patients
**BFI**	Dearden et al., 2019 (55)	Survey in patients with mCRPC who had been taking abiraterone or enzalutamide treatment for ≥2 months (2L+/N=152)	Mean BFI score • Overall cohort (n=152): 3.2 • Abiraterone (n=78): 2.9 • Enzalutamide (n=74): 3.6
	Stenner et al., 2018 (64)	Swiss multicenter registry was prospective, longitudinal, noninterventional study in patients with mCRPC which had progressed after 1L treatment with docetaxel (2L/N=138)	Fatigue Score, mean ± SD Overall cohort (n=138) • Visit 1 (n=136): 3.69 ± 2.28 • Visit 2 (n=122): 3.35 ± 2.39 • Visit 5 (n=95): 2.97 ± 2.23 • Visit 7 (n=80): 3.23 ± 2.44
**AQoL-8D**	Parente et al., 2017 (53)	Patients with mCRPC previously treated with a docetaxel-containing regimen treated with cabazitaxel (25 mg/m^2^) every 3 weeks plus prednisone/prednisolone (10 mg daily)	Overall score, mean ± SD • Baseline (n=104): 0.70 ± 0.21 • Cycle 2 (n=96): 0.73 ± 0.19 • Cycle 4 (n=80): 0.77 ± 0.17 • Cycle 6 (n=63): 0.76 ± 0.17 • Cycle 10 (n=32): 0.79 ± 0.17 • EOT (n=50): 0.68 ± 0.20

*EQ-VAS is measured on a scale from 0 to 100, with higher values indicating better HRQoL.

^†^Clinically meaningful improvement of Total FACT-P was defined as a minimal change of 10 points from baseline for the Total FACT-P score.

^‡^The clinically meaningful improvement of BPI-SF score (CMC-BPI) was defined as a change of score of at least 30% from baseline score, with a minimum of 2 points.

^§^BPI-SF severity is measured on a scale from 0 to 10, with higher scores indicating less pain severity.

^¶^95% CI excludes zero, indicating a statistically significant change in TAMC.

1L, first line; 2L(+), second line (and beyond); AQoL-8D, Australian Quality of Life-8 dimension questionnaire; BFI, Brief Fatigue Inventory; BPI-SF, Brief Pain Inventory-Short-form; C20, cabazitaxel 20 mg/m^2^; C25, cabazitaxel 25 mg/m^2^; D75, docetaxel 75 mg/m^2^; CI, confidence interval; CRPC, castration-resistant prostate cancer; CT, chemotherapy; EAP, Early Access Program; EORTC QLQ-INFO25, European Organization for Research and Treatment of Cancer Quality of Life Questionnaire Information Needs, 25-item questionnaire; EOT, end of treatment; EQ-5D, EuroQol 5 dimensions questionnaire; EQ-5D-3L, EuroQol 5 dimensions questionnaire 3 level; EQ-5D-5L, EuroQol 5 dimensions questionnaire 5 level; EQ-5D VAS, EuroQol 5 dimensions visual analogue scale; ES, effect size; FACT-G, Functional Assessment of Cancer Therapy-General; FACT-P, Functional Assessment of Cancer Therapy-Prostate; HRQoL, health-related quality of life; IQR, interquartile range; LS, least squares; mCRPC, metastatic castration-resistant prostate cancer; MCS, mental component score; PC, prostate cancer; PCS, physical component score; Q(1/3), question (1/3); SD, standard deviation; SE, standard error; SF-36, 36-item short-form health survey; SRE, skeletal-related event; SSE, symptomatic skeletal event; TAMC, trajectory-adjusted mean change; TIBI-Cap, total illness burden index for prostate cancer.

There were no clear trends for associations between improvement in HRQoL and mCRPC treatment. EQ-5D was either maintained or improved with treatment among populations that were diverse with respect to prior treatment history, irrespective of the treatment type (cabazitaxel, abiraterone, enzalutamide, docetaxel) (**Table 4**) (49, 55–58). Two studies demonstrated clinically important improvements or minimally important differences (change in visual analog scale score of ±7 points from baseline) after treatment with 2L cabazitaxel or 1L or 2L enzalutamide (49, 58).

Overall, HRQoL, as measured using FACT-G/P, was found to be either maintained or improved by treatments (including Ra-223, cabazitaxel, abiraterone, enzalutamide, docetaxel) in 55%–80% of patients (**Table 4**) (49, 58, 59, 61, 64, 65). Clinically relevant improvements in scores of FACT-P or its subscales, including physical well-being, social well-being, emotional well-being, functional well-being, and prostate-specific concerns, were also observed in some studies (**Table 4**) (58, 60, 65). There was some variation in the definitions of these improvements between the studies (ranging from ≥6 points to ≥16 points for FACT-P); the recommended range for clinically meaningful change is 6 to 10 points (67). For example, one registry-based study of heavily pretreated patients (including abiraterone and/or enzalutamide, docetaxel, cabazitaxel, or radiotherapy) with mCRPC noted a clinically meaningful improvement in FACT-P total score, defined as a change of 10 points from baseline in 31.4% of all patients and in 37.7% and 26.7% of those with and without pain at baseline, respectively, after treatment with Ra-223 (60). Another registry study of docetaxel-pretreated patients who received 2L therapies in Switzerland reported a low rate of improvement in the FACT-P total score of 23%, which they attributed potentially to the rather high threshold definition of a “clinically meaningful change” of ≥16 points (64). In yet another study evaluating patients from the PROSELICA (2L) and FIRSTANA (1L) trials, the threshold for a “definitive improvement from baseline” was ≥7 (65). In addition to different thresholds for clinically relevant findings, interpretation of the data should take into consideration the size, and the disease and treatment characteristics of the patient populations, as well as the widely varied treatments provided between these studies. SSEs are reported to have a substantial and negative impact on the HRQoL of patients with bone-metastatic mCRPC, with lower FACT-P functional well-being scores compared with non-SSE cohorts (**Table 4**) (2, 62).

Data for BPI-SF varied across studies, again likely due to differences between studies with regard to the patient population and design (**Table 4**). In a registry study of patients scheduled for Ra-223 treatment, 31.4% achieved a complete pain response (defined as a score of 0 on the BPI-SF “worst pain” item and no increase in daily use of analgesics). However, a clinically meaningful improvement in BPI-SF worst pain during treatment (defined as ≥30% change from baseline) was achieved by 49.5% of patients overall, and by 77.7% of those with pain at baseline (60). In the UK EXTREQOL trial, 40%–43% and 58%–65% of patients with moderate/severe pain at baseline saw clinically meaningful improvements (defined as a ≥2 point change in BPI-SF from baseline) in pain severity and interference, respectively, during systemic treatment for mCRPC (59). Findings were similar for the European PREMISE trial (observational study of enzalutamide in mCRPC), in which the same threshold for clinically meaningful change was used for pain severity, while that for pain interference was 1.25 (58). The UK EXTREQOL was the only study to use EORTC QLQ as a tool to assess humanistic burden. After 6 months of treatment, the mean change in global score from baseline was –1.01 (95% confidence interval [CI], –3.57 to 1.57; *P =* 0.44) (59).

### 3.4 Economic burden

Of the 456 full-text papers screened, 74 were included from 74 original studies, comprising 20 cost-effectiveness analysis (CEA)/cost-utility analysis studies, two budget impact models (BIMs), 15 HTA reports, and 37 studies reporting costs/healthcare resource use (HCRU). A total of nine records were finally included in this manuscript: eight papers and one HTA (**Supplemental Figure S5**; **Tables 5**, **6**).

**Table 5:** Summary of included studies reporting total costs.

Author	Study design	Country/currency	Sample size	Study population	Cost year	Total costs
Healthcare resource use studies						
Kreis et al., 2021 (68)	Claims study	Germany/Euro	3944	Patients with mCRPC treated with cabazitaxel (N=240), docetaxel (N=539), abiraterone (N=486), enzalutamide (N=351), and BSC (N=2328)	2014–2017	Mean all-cause/mCRPC-related monthly costs Cabazitaxel: €7631/€6343 Abiraterone: €5226/€4579 Enzalutamide: €5079/€4416 Docetaxel: €2392/€1580 BSC: €959/€438
Schultz et al., 2018 (69)	Retrospective study	USA/USD	3230	Patients with mCRPC treated with enzalutamide (N=847) or abiraterone (N=2018)	2017	All cause total healthcare monthly cost (mean ± SD): Enzalutamide: $14,934 ± $12,391 Abiraterone: $14,961 ± $16,094 PC-related total healthcare monthly costs (mean ± SD): Enzalutamide: $11,598 ± $7974 Abiraterone: $10,975 ± $12,051
Restelli et al., 2017 (70)	Observational study	Italy/Euro	9700	Patients with mCRPC	2016	Total cost for 1 year for mCRPC management: €196.5 to €228 million 1L treatment: €136.9 to €160.3 million 2L treatment: €59.7 and €67.8 million
Cost-effectiveness studies						
Barqawi et al., 2019 (71)	CEA	USA/USD	NR	Patients with mCRPC resistant to docetaxel therapy	2019	Lifetime costs (abiraterone): $115,433 Lifetime costs (cabazitaxel): $85,377 Lifetime costs (enzalutamide): $109,213
Zhang et al., 2021 (72)	CEA (CARD trial)	USA/USD	NR	Patients with mCRPC receiving cabazitaxel	2021	Total costs (cabazitaxel): $105,169.70 Total costs (enzalutamide/abiraterone): $55,682.67
Su et al., 2020 (73)	CEA (PROfound trial)	USA/USD	387	Patients with mCRPC who have progressed on prior hormonal agent	2019	Total costs (olaparib, 3 gene alterations): $24,626 Total costs (olaparib, 15 gene alterations): $48,526 Total costs (enzalutamide/abiraterone, screened for up to 3 gene alterations): $17,243 Total costs (enzalutamide/abiraterone, screened for up to 15 gene alterations): $55,476
Li et al., 2021 (74)	CEA	USA/USD	NR	Patients with mCRPC whose disease progressed during enzalutamide or abiraterone treatment	2020	Base case analysis: (enzalutamide or abiraterone vs. olaparib): Total cost enzalutamide: $80,154 Total cost olaparib: $157,723

1L, first line; 2L, second line; BSC, best supportive care; CEA, cost-effectiveness analysis; mCRPC, metastatic castration-resistant prostate cancer; NR, not reported; OS, overall survival; PC, prostate cancer; SD, standard deviation; USD, United States Dollar.

**Table 6:** Summary of included studies reporting economic evaluations.

Author	Study design	Country/perspective	mCRPC population	Interventions	ICER	QALY
Cost-effectiveness studies						
Barqawi et al., 2019 (71)	CEA	US healthcare payer	Visceral mCRPC resistant to docetaxel	Cabazitaxel, enzalutamide, abiraterone	ICER/LY (cabazitaxel vs. abiraterone): dominant ICUR/QALY (cabazitaxel vs. abiraterone): $1.5 million ICER/LY (enzalutamide vs. cabazitaxel): $238,362 ICUR/QALY (enzalutamide vs. cabazitaxel): $103,636	QALYs (abiraterone): 0.58 QALYs (cabazitaxel): 0.56 QALYs (enzalutamide): 0.79
Li et al., 2021 (74)	CEA	US payer perspective	Patients with mCRPC who progressed during treatment with enzalutamide or abiraterone	Olaparib, enzalutamide + abiraterone	Olaparib: $248,248/QALY	Total QALY (enzalutamide + abiraterone vs. olaparib): 0.95, 1.26
Su et al., 2020 (73)	CEA	US payer perspective	Patients with mCRPC who progressed on prior hormonal agent (PROfound trial)	Olaparib vs. enzalutamide or abiraterone	ICER/QALY (olaparib vs. enzalutamide or abiraterone, 3 gene alterations): $116,903 ICER/QALY (olaparib vs. enzalutamide or abiraterone, 15 gene alterations): dominant	QALY (olaparib, 3 gene alterations): 0.173 QALY (olaparib, 15 gene alterations): 0.380 QALY (enzalutamide or abiraterone, 3 gene alterations): 0.109 QALY (enzalutamide or abiraterone, 15 gene alterations): 0.313
Zhang et al., 2021 (72)	CEA	US payer perspective	Patients with progressive mCRPC treated with ≥3 cycles of docetaxel (CARD trial)	Cabazitaxel vs. enzalutamide or abiraterone	ICER/QALY (cabazitaxel vs. enzalutamide or abiraterone): $309,294/QALY	QALY (cabazitaxel): 0.57 QALY (enzalutamide or abiraterone): 0.41 QALY: cabazitaxel vs. enzalutamide or abiraterone: 0.16
Budget impact models						
Flannery et al., 2017 (75)	BIA	US managed care plan	Prior docetaxel treated mCRPC	Cabazitaxel, abiraterone, enzalutamide, Ra-223	NR	NR
HTA						
SMC Scotland (76)	HTA	SMC Scotland, NHS	Adult patients with mCRPC and *BRCA1/2* mutations (germline and/or somatic) who progressed following prior therapy that included a new hormonal agent	Olaparib vs. cabazitaxel and docetaxel	Cabazitaxel: £3296 Docetaxel: £55,957	Olaparib: 1.61 Cabazitaxel: 0.73 Docetaxel: 0.73

BIA, budget impact analysis; *BRCA1/2*, breast cancer susceptibility genes *BRCA1* and *BRCA2*; CEA, cost-effectiveness analysis; CT, chemotherapy; HTA, health technology assessment; ICER, incremental cost-effectiveness ratio; ICUR, incremental cost-utility ratio; LY, life-year; mCRPC, metastatic castration-resistant prostate cancer; NHS, National Health Service; NR, not reported; QALY, quality-adjusted life-year; Ra-223, radium-223; SMC, Scottish Medicines Consortium; US, United States.

Most (n=6) of the papers were from the US perspective. Seven publications reported on the costs associated with mCRPC, including three HCRU papers and four CEA studies (**Table 5**), and six publications reported economic evaluations in mCRPC (**Table 6**). There was one BIM and one HTA that reported an economic evaluation in mCRPC from a UK (Scottish) perspective (**Table 6**).

#### 3.4.1 Costs

Three HCRU studies, one observational study from each of Italy and Germany, one retrospective study from the US, and four CEA studies, all from the US perspective, were included, comparing the cost of abiraterone, enzalutamide, olaparib, best supportive care, docetaxel, and cabazitaxel (**Table 5**). Reflective of real clinical practice, the analysis by Restelli et al. (70) in Italy demonstrated the high economic cost of mCRPC. Annual direct medical costs ranged from €196.5 million to €228.0 million, representing ~0.2% of the financing of the Italian National Health Service in 2016 (70). Overall, the annual cost of 1L treatment in Italy is more than twice that for 2L at €136.9–160.3 million versus €59.7–67.8 million. In large part, this difference is probably reflecting the drop in patient numbers receiving 2L treatment (6497 vs. 3203) (70). Few studies have reported the cost of genetic testing. However Su D et al. (2020) reported a cost of $5800 for next-generation sequencing (73).

The total cost of mCRPC is also high in the US, as reported by cost-effectiveness modeling studies, with costs differing by treatment regimen and tumor characteristics. Costs for patients treated with olaparib, enzalutamide, or abiraterone increased substantially in the subgroup of patients with a tumor harboring at least one of three prespecified gene alterations (*BRCA1*, *BRCA2*, or *ATM*) versus those with at least one of 15 prespecified gene alterations (**Table 5**). This outcome was thought to be at least in part attributable to the greater number of patients that would need to be screened (and thus greater cost of screening) if only three specific gene alterations are sought compared with if any of 15 gene alterations are sought to identify patients eligible for treatment (73). In addition, since OS in patients with alterations in genes other than *BRCA1*, *BRCA2*, or *ATM* was comparable between those treated with olaparib versus standard of care (46), it could be that treating these patients with the relatively lower-cost olaparib would require fewer health resources toward similar outcomes (73).

The available data from direct comparisons have demonstrated that olaparib is generally more costly than abiraterone/enzalutamide, particularly when patients are screened only for mutations in *BRCA1*, *BRCA2*, or *ATM* (**Table 5**) (73, 74). Findings across studies indicate that the costs of treatment with enzalutamide and abiraterone are similar, and are lower than for cabazitaxel (69, 71, 72). Among these three treatment options, one study in the US concluded that enzalutamide was the preferred treatment option from a clinical and healthcare payer perspective. Over a lifetime (5-year horizon model), enzalutamide provided better life-years and QALY outcomes than either abiraterone + prednisone or cabazitaxel + prednisone at a lower cost than for abiraterone and higher than for cabazitaxel (US$109,213 vs. US$115,433 and US$85,377, respectively) in patients with post-docetaxel therapy resistance (71). Limitations of these findings include the different study designs, that all but two of the studies were conducted in the US, and that they were obtained in different patient populations with respect to sample number – either not reported (71, 72, 74) or ranging from 387 to 9700 (69, 70, 73) – and prior treatment history.

#### 3.4.2 Economic evaluations

Six economic studies reporting ICERs and/or QALYs were included in this report: four CEA studies, one BIM, and one HTA (**Table 6**). All of the CEA studies were from the US, and the HTA was from the UK (SMC). One study found that enzalutamide provided greater QALYs than either abiraterone or cabazitaxel for patients with mCRPC and visceral involvement after docetaxel therapy resistance. Enzalutamide was cost-effective from a US payer perspective at 92% of the time with a willingness-to-pay (WTP) threshold of $100,000/QALY (71). Cabazitaxel was not cost-effective in patients enrolled in the CARD trial (72); however, this is based on only one study. According to the SMC, olaparib was cost-effective compared with both docetaxel and cabazitaxel in patients with mCRPC and *BRCA1* or *BRCA2* mutations (germline and/or somatic) who had progressed following prior therapy that included an ARPI (**Table 6**) (76). There were conflicting results for the cost-effectiveness of olaparib in the US: one study determined that olaparib was not cost-effective (in line with common WTPs in the US), with an ICER of $248,248 per QALY gained (**Table 6**) (74), whereas another study reported it was cost-saving, with a cost per QALY of $116,903 (73). However, the authors concluded that olaparib was a preferred option in patients with any of 15 prespecified genes (**Table 6**) (73).

## 4 Discussion

The findings of these structured and systematic literature reviews demonstrate the challenges associated with the limited alternatives available for the treatment of mCRPC in the ≥2L setting. The data establish the existence of both a humanistic and economic burden in ≥2L mCRPC; patients are heavily affected in terms of HRQoL, side effects, and survival, as well as incurring substantial healthcare costs. That said, this burden is not limited to the ≥2L setting. Many patients do not receive treatment beyond 1L, and published data collected in the 2010s show that most patients with ≥2L mCRPC will receive only palliative care (77–80), contrasting starkly against the number of therapies being studied in phase 3 trials. There is a lack of data informing decisions around optimal treatment sequencing, including how clinicians and patients make these decisions while taking into account cost-effectiveness. Thus, there is a clear unmet need in patients with mCRPC who have been treated with at least one ARPI and at least one taxane. International collaboration is needed to standardize treatment sequencing to provide superior outcomes for these patients.

The information collected in this review regarding treatment patterns in mCRPC in the recent epoch before the introduction of PARP inhibitors in 2020 is generally confirmed by recently published real-world studies from the US, Australia, Europe, and Japan (9, 81–83). The ARPIs enzalutamide and abiraterone were the most common 1L systemic options for patients with mCRPC; the next most frequently used 1L systemic option was chemotherapy (primarily docetaxel), and then sipuleucel-T (9, 81, 83). Of note, one study reported at congress (thus not included in the review) noted undertreatment of patients in the US, with 38% of patients not receiving any 1L therapy (78). Similar findings regarding treatment patterns were reported in Canada and Australia (also congress reports), with the exception of sipuleucel-T; the most commonly used 1L options were ARPIs (26%–94%) and docetaxel (26%–30%) (84–86). In Spain, 64.2% of patients who had mCRPC with DDR gene mutations were treated in the 1L setting with abiraterone plus prednisone/enzalutamide (87).

The preferred 2L systemic option was chemotherapy in all countries except Japan, where either enzalutamide (83) or LHRH agonists/antagonists (44) were the more common options, and Spain, where ARPIs and chemotherapy were used equally often (83). In one study in Spain that was reported in a congress abstract, the most frequent choice of 2L therapy was abiraterone/enzalutamide (31.4%) or docetaxel (30.6%); however, 24.8% received no 2L therapy (87). Docetaxel is reportedly more commonly used as a 2L option in Canada (57%) compared with the US (6%–22%). Overall, these findings are consistent with National Comprehensive Cancer Network (NCCN) guidelines (12). Combination therapy with olaparib and abiraterone is currently being studied as a 1L option in the phase 3 PROpel trial, with recently published data demonstrating significantly improved rPFS over abiraterone alone in patients with mCRPC (25.0 vs. 16.4 months; HR 0.67; 95% CI 0.56–0.81), regardless of whether or not they harbored HRR gene mutations. OS data were immature in the planned interim analysis (40%); however, a trend toward improved OS with olaparib plus abiraterone versus abiraterone alone was reported (HR 0.83; 95% CI 0.66–1.03) (88). The HRs for time to first subsequent therapy or death and time to second progression or death (0.74 and 0.69, respectively), reported in an earlier primary analysis of PROpel, supported efficacy beyond first imaging-based progression (26). In August 2023, niraparib (not yet approved for prostate cancer) was approved in combination with abiraterone for the treatment of patients with deleterious or suspected deleterious *BRCA1*- or *BRCA2*-mutated mCRPC, based on the findings of the MAGNITUDE trial (NCT03748641) (89). This combination is also under investigation for the treatment of patients with deleterious HRR gene-mutated metastatic castration-sensitive prostate cancer (AMPLITUDE; NCT04497844) (89). It is also being studied in combination with cetrelimab (anti-programmed death 1 antibody) in patients with mCRPC, including the subpopulation with DDR gene defects (QUEST; NCT03431350) (90, 91), regardless of prior treatment history (except for prior PARP inhibition in AMPLITUDE).

There appears to be more variation regarding treatment sequencing, the importance of which has been emphasized in the updated Prostate Cancer Working Group recommendations (92, 93). There is currently no evidence-based guidance or consensus on the optimal sequence beyond 2L, and a lack of randomized trials to clarify the issue. According to a recent PubMed-based literature review of clinical studies on treatment sequencing and combinations in mCRPC, abiraterone followed by enzalutamide was the best sequential treatment in docetaxel-naïve patients, whereas enzalutamide was the most effective subsequent choice of treatment for patients who had previously failed on docetaxel (94). The findings of a phase 2 crossover study with HRQoL as an outcome measure support the use of abiraterone as a first subsequent therapy in patients with treatment-naïve mCRPC (95). In the CARD study, patients with mCRPC who had failed on docetaxel and either abiraterone or enzalutamide (before or after docetaxel), and then treated with 3L cabazitaxel experienced longer rPFS and OS than their counterparts who were treated in 3L with the other ARPI (i.e., abiraterone in those who had previously received enzalutamide, and vice versa) (47). The most common 1L-to-2L sequence identified in the present literature review was ARPI-to-chemotherapy, with other sequences being chemotherapy-to-ARPI, ARPI-to-ARPI, and chemotherapy-to-chemotherapy. Several trials are ongoing with ARPI plus androgen deprivation therapy (ADT) in combination with a third agent (“triplet therapies”) to treat metastatic hormone-sensitive prostate cancer (mHSPC), such as ENZAMET, ARASENS, and PEACE-1. Findings to date provide equivocal evidence that (at least for patients with high-volume mHSPC) OS, PFS, and various measures of PROs are improved by the addition of agents such as docetaxel, darolutamide, enzalutamide, and abiraterone to ADT (96–100). These findings may lead to changes in mCRPC sequencing. However, it should be noted that the timing of administration of the docetaxel-related regimen differed between the three trials (i.e., delivered before, during, or just after ARPI) (97, 98, 100). Furthermore, trials are underway to identify molecular biomarkers that predict response or primary resistance to particular treatments, which may be invaluable in the treatment decision-making process to optimize the patient’s therapeutic journey (101, 102). Biomarkers currently under study include: circulating androgen receptor gene status (assessed using liquid biopsy); alterations in phosphatase and tensin homolog protein and its pathway; immune biomarkers (e.g., programmed death ligand 1, sex-determining region Y-box 2; and DDR gene mutations (103–105). The multicenter, outcome-adaptive, biomarker-driven ProBio study is currently using a biomarker-driven platform to inform treatment decisions in men with mCRPC (102).

Geographic differences in the preferred treatment sequence were noted; for example, chemotherapy-to-ARPI was a more common 1L-to-2L sequence than ARPI-to-ARPI and chemotherapy-to-chemotherapy in Germany, Italy, and the UK, while chemotherapy-to-ARPI and ARPI-to-ARPI was administered to a similar degree in France and Spain (83). The most frequent 1L-to-2L treatment sequence in a large retrospective study in the US was abiraterone-to-enzalutamide, followed by enzalutamide-to-abiraterone (82), while in Ontario, Canada, the preference was to provide a subsequent (2L) treatment with a different mechanism of action (abiraterone or enzalutamide followed by docetaxel, with Ra-223 being the most common 3L treatment) (80). A retrospective, multicenter analysis of real-world data performed in Australia found that the most frequent subsequent systemic therapy was a carboplatin-based regimen, and that some patients also received rechallenge with an ARPI or docetaxel (106). However, it was also reported that choice of subsequent treatment did not impact survival outcomes; median OS did not differ according to subsequent systematic therapy (106). In contrast, in a single-center study, docetaxel rechallenge has shown meaningful anti-tumor activity in pretreated patients with mCRPC in the salvage setting (107).

Geographic differences in treatment sequencing preference may be attributable to a variety of country-, patient-, and physician-based factors, such as whether or not ARPI rechallenge is allowed, reimbursement rules, patient preference for oral versus intravenous agents, access and cost of newer agents, and the specialism of the treating physician (83, 108–110). Patients may live in regions without access to newer agents or where screening is not common and they are diagnosed at a later stage, rendering them ineligible for certain therapies (44, 108). Rechallenge with ARPIs is discouraged in ESMO and European Association of Urology (EAU) guidelines due to the potential for cross-resistance (101, 111, 112), but in the local guidelines for Germany, Italy, and Japan it is a suggested treatment option (83). Moreover, local reimbursement criteria may preclude rechallenge with a different ARPI – this is the case in Canada, with exceptions in the case of intolerance with the first ARPI or (in some provinces) if chemotherapy was used in between (“sandwich therapy”) (80, 113). According to one US study that compared therapy choice between oncologists and urologists, a significantly greater proportion of patients treated by oncologists were prescribed hormone therapy, chemotherapy, and radiation therapy; nonchemotherapy options were significantly more commonly prescribed by urologists than by oncologists (114). Another study from the US reported that in the 2L setting, oncologists were most likely to prescribe hormone therapy and chemotherapy, while for urologists the most commonly prescribed treatments were sipuleucel-T and hormone therapy (115). Other work from the US reported that urologists were increasingly prescribing oral therapies such as abiraterone and enzalutamide (116). Treatment options for mCRPC continue to evolve, and particularly in the 2L setting with the recent approval of several novel therapies such as PARP inhibitors (olaparib, rucaparib), immunotherapies (pembrolizumab), and ^177^Lu-PSMA-617. Although all of these new agents are now included in one or more of the recently updated NCCN, ESMO, EAU, and American Urological Association prostate cancer treatment guidelines, there is little real-world evidence regarding their use (12, 110, 111, 117). Olaparib and rucaparib are both approved in one or more countries (including the US) for the treatment of patients with HRR gene-mutated mCRPC who have progressed on ARPIs (olaparib) or in patients with mutations in *BRCA1* or *BRCA2* who have progressed on an ARPI and a taxane-based chemotherapy (rucaparib). Of note, NCCN guidelines do not recommend use of olaparib for patients harboring *PPP2R2A* mutations due to preliminary evidence of reduced efficacy in these patients (12). For both of these PARP inhibitors, gene mutations are confirmed using US Food and Drug Administration-approved companion diagnostics (18, 19). Among patients with mCRPC, only 11%–33% harbor germline mutations in DDR genes, including HRR genes such as *BRCA1*/*BRCA2* (118, 119); therefore, the majority of patients are not eligible to receive them. Moreover, in a recent network analysis of the PROfound and CARD studies, it was noted that certain patient subgroups achieved reduced or no benefit with olaparib, including those harboring mutations in genes other than *BRCA1* or *BRCA2*, compared with active standard-of-care agents (e.g., cabazitaxel) (120). Pembrolizumab is also approved in the US for the treatment of patients with mismatch-repair-deficient or microsatellite-instability-high solid tumors who have no satisfactory alternative (21), and ^177^Lu-PSMA-617 is approved for patients with PSMA-positive (determined using a PSMA imaging agent) mCRPC who have progressed on an ARPI and taxane-based therapy (121). It remains to be seen how these new agents will be incorporated into the treatment landscape in the real-world setting. Access to all therapy options has been shown to result in longer survival in patients with prostate cancer (122).

Most pivotal phase 3 trials for currently approved treatments for mCRPC were published prior to 2017 (**Supplemental Table 15**), and thus data for only two trials were included in this review: PROfound (olaparib vs. enzalutamide plus abiraterone) (45, 46) and CARD (cabazitaxel vs. enzalutamide or abiraterone plus prednisone) (47). Despite differences in the patient populations, both studies demonstrated improved survival with the study drug over abiraterone or enzalutamide. In PROfound, OS varied (according to the particular cohort) from 14.1 to 19.1 months with olaparib and from 11.5 to 15.1 months with an ARPI; rPFS was 5.8 versus 3.5 months (45, 46). In CARD, OS was 13.6 months with cabazitaxel versus 11.0 months with an ARPI, and PFS was 4.4 and 2.7 months, respectively (47). Based on these clinical trials, newer agents have been approved and brought into clinical practice (12, 112). Survival data for other approved treatments that were outside the scope of this review are provided in **Supplemental Table S15**.

The humanistic burden associated with mCRPC was high across the included studies, demonstrating that mCRPC is associated with poor HRQoL and imposing a significant burden on patients. This burden was worse for patients with SSEs, skeletal-related events, or bone metastases (2, 62). Although treatment was often associated with a delayed deterioration in HRQoL, no trends for one regimen over another emerged. Two studies (not identified in the current review) determined that patients treated with abiraterone reported less fatigue and cognitive impairment and had more favorable PROs compared with enzalutamide in both real-world and clinical trial settings (95, 123). However, a propensity score-matching study found that whilst there was no significant difference in the development of new comorbidities between the two agents, OS was better with enzalutamide; moreover, the overall Charlson Comorbidity Index score (and not its items) was a significant predictor of OS (124). There is increasing awareness that HRQoL is an important endpoint of value to both patients and healthcare providers. While the impact of treatments on many aspects of HRQoL in patients with localized disease is well established, there is less information on patients with advanced disease who are more likely to have poor performance status, are more frequently affected by pain due to bone metastases, and for whom the balance between longevity and quality of life is perhaps more important (125, 126). A recent systematic review of patient values, preferences, and expectations found that, while cancer progression or survival, pain, and fatigue were key considerations regarding treatment decisions, alleviation of pain was valued at the expense of survival benefits among symptomatic patients (127). PRO endpoints are included in clinical registration trials, as well as product labels of the latest interventions, and form an important part of disease management in patients with mCRPC (128, 129). However, the tools used to measure HRQoL in patients with mCRPC may not fully capture patients’ lived experiences of the burden of advanced prostate cancer (130–132). Some HRQoL instruments (e.g., EORTC QLQ-C30) are generic and have not been optimized to measure the specific burden of mCRPC; prostate cancer-specific tools (e.g., FACT-P) may capture a more accurate picture. It is therefore encouraging that a recent systematic review of trials on advanced prostate cancer conducted between 2011 and 2019 found that FACT-P was the most frequently adopted HRQoL tool (11/14 trials), followed by EQ-5D (6/14 trials) and EORTC QLQ-C30 (3/14 trials) (129). The evidence from that review demonstrated that in phase 3 trials, currently available systemic treatments (including ARPIs, Ra-223, and docetaxel) had a positive impact on HRQoL in patients with advanced cancer compared with standard ADT. Data for newer agents such as olaparib were available only for phase 2 trials, but nonetheless were also found to be beneficial (129). Moreover, some instruments are more meaningful at particular stages of the disease. Holmstrom et al. describe a conceptual model that characterizes patient experiences of living with mCRPC, indicating symptoms and impacts, such as fatigue, pain, urinary frequency, interference with daily activities, and frustration, that are not adequately assessed with currently available PRO instruments (133).

Further studies are required to determine the cost-effectiveness of the recently approved therapies, the optimal treatment sequence in patients who have progressed, and models that use data from real-world studies, not clinical trials. The economic burden to healthcare systems of patients with mCRPC is ongoing as patients are still progressing on currently available treatments.

There is a lack of overall consensus on genetic testing. US and European guidelines now recommend genetic testing (somatic and/or germline), especially in patients with advanced prostate cancer, and earlier genetic testing in patients who are high risk (including those with a relevant family history) (134, 135). In contrast, a post-meeting survey from the Asia-Pacific Advanced Prostate Cancer Consensus Conference revealed that biomarker testing is limited to patients who have progressed on multiple lines of therapy (136).

In summary, the lack of available data to inform the delivery of appropriate treatments beyond 2L to patients with mCRPC means that the optimal sequence remains undefined (85, 106, 137). The data have shown that options with alternative mechanisms of action may be preferred to avoid cross-resistance between ARPIs (80, 101). The treatment options for mHSPC are increasing, and future real-world studies assessing the impact of mHSPC treatment algorithms on subsequent mCRPC treatment alternatives are needed. In addition, there is a need for innovative treatment options in post-taxane and post-ARPI mCRPC that improve survival and HRQoL, as well as having a tolerable safety profile. Aligning and then switching from 1L to 2L mCRPC therapies in a timely manner will help to ensure patients receive the most benefit from a clinically efficacious agent. New treatment options for the ≥2L setting may help not only treatment sequencing optimization, but may also result in improvements to the humanistic and economic burden.

### 4.1 Key gaps in current literature and suggestions for future research

The literature search identified only seven HCRU/cost-effectiveness studies and six studies on treatment patterns from 2009 to 2021, among which five and three, respectively, originated in the US. In addition, only one study provided a cost associated with genetic testing (73). There is a clear need for studies analyzing both cost and treatment patterns across diverse geographic regions to provide a clear view of the barriers to treatment access.

### 4.2 Strengths and limitations

The strengths of this review include the comprehensive search of recent literature over multiple databases for studies describing the global treatment landscape and disease burden associated with mCRPC. The methodology utilized both structured/targeted reviews and systematic literature reviews, and searches were repeated to ensure up-to-date information was captured. This strategy has allowed us to meet our objective of identifying unmet needs for patients with late-stage mCRPC, demonstrating the overall high disease burden, the need to establish optimal treatment sequencing, and the global need to develop novel treatments that are affordable, well tolerated, and can be implemented using a holistic approach.

One key limitation of this study is the heterogeneity between studies with regard to the characteristics of the patient population, study design, the tools used to measure PROs, and the terminology used to describe the therapies (e.g., enzalutamide and abiraterone are referred to variously in the literature as novel/new hormonal agents, ADT, ARPIs, and androgen receptor inhibitors). In addition, the search strategy was limited by the exclusion of some phase 2 or 3 trials of key approved therapies (olaparib, rucaparib, pembrolizumab), which means that the data on current treatment options may not be representative, and by the inclusion of only English-language publications from the past 5 years. Furthermore, it was not possible to explore the effect of race/ethnicity, since details of these were not provided in many of the identified studies (these are detailed in recent congress publications, and were therefore outside the scope of this review).

Finally, caution is needed when reviewing the cost-effectiveness data, since the therapies involved have different indications, eligible patient populations, and prior therapy requirements.

### 4.3 Conclusions and future directions

The findings outlined in this report highlight a lack of treatment options with novel mechanisms of action and more tolerable safety profiles that meet patient needs and preferences. Treatment approaches that improve survival and HRQoL are needed, ideally while simultaneously reducing costs and HCRU. The last decade has seen a renaissance in mCRPC treatment options, with several life-prolonging therapies becoming established. It is apparent from this literature review that treatment sequencing and patient follow up is not homogenous, resulting in varied patient outcomes by region, healthcare system, and physician. Further prospective studies should be conducted to define the optimal treatment sequence and time to change treatment. During their treatment journey, patients should expect to undergo germline and somatic genetic testing to allow strategic implementation of PARP inhibition or immune checkpoint inhibition with pembrolizumab. Further improvements are signaled by the advent of PSMA radioligand imaging, which promises to improve the diagnosis of prostate cancer. Moreover, its routine implementation in the clinic could provide a basis for selection of patients with mCRPC who may respond to novel PSMA-targeted radioligand therapy. All patients should also receive holistic care that provides psychosocial support, attention to physical function and frailty prevention, and caregiver support to ensure optimal outcomes for all patients and timely, informed treatment decisions.

## Author contributions

OM and RG designed this study. GK contributed to the literature search, review, and data extraction. DL, SC, AM, OM, and RG contributed to manuscript drafting and manuscript revision. All authors have reviewed and approved the final version of this manuscript.
