# Supplementary material for: Treatment landscape and burden of disease in metastatic castration-resistant prostate cancer: systematic and structured literature reviews
Source: Front Oncol. 2023 Sep 27;13:1240864. doi: 10.3389/fonc.2023.1240864 (PMC10565658; doi:10.3389/fonc.2023.1240864)
Supplement: Supplementary file 1 [file DataSheet_1.pdf]

SUPPLEMENTAL INFORMATION

SUPPLEMENTAL FIGURE S1

Methodology for structured reviews

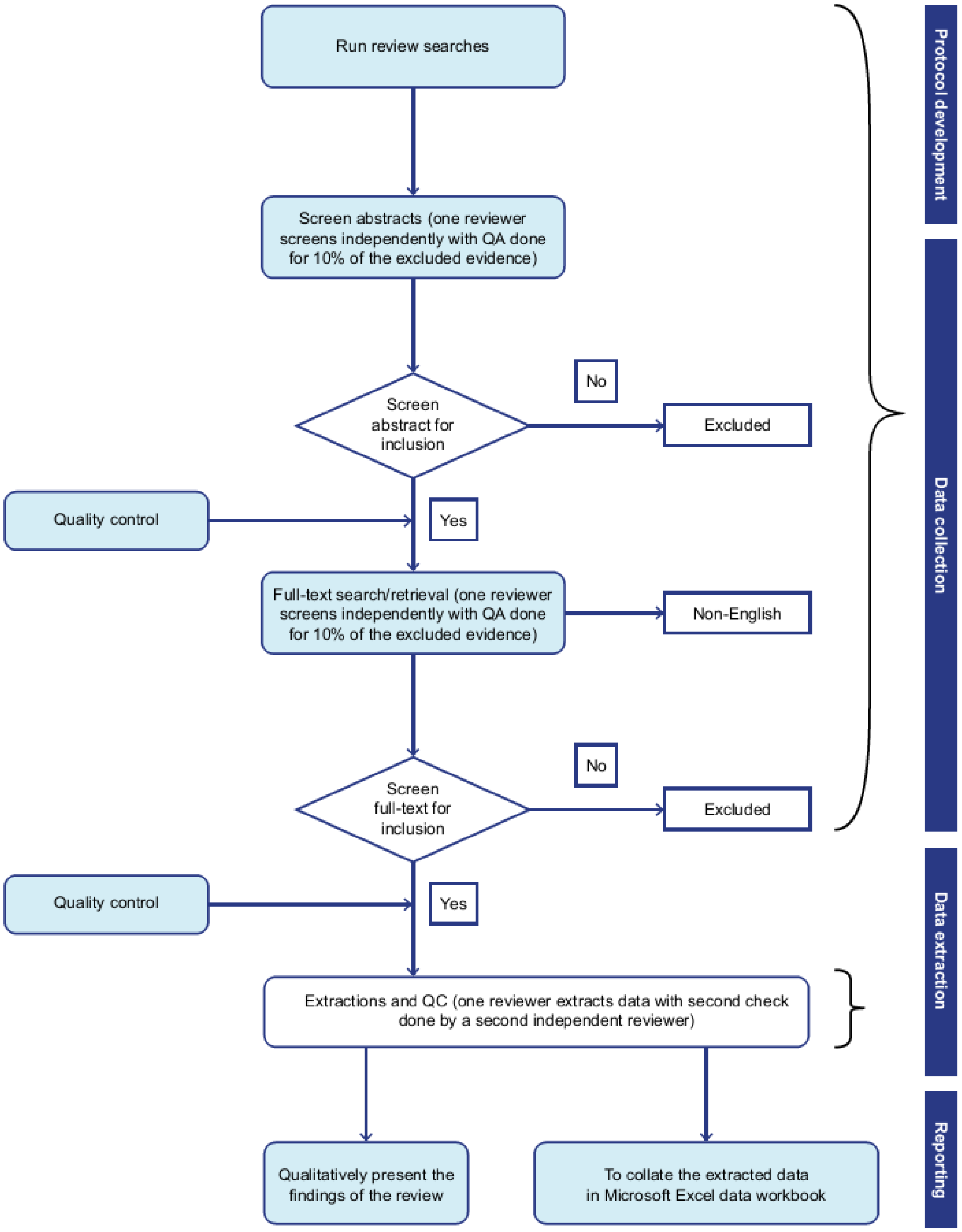

QA, quality assurance.

SUPPLEMENTAL FIGURE S2

Flow of studies included in the structured literature review of guidelines, treatment landscape, and safety of metastatic castration-resistant prostate cancer (original structured reviews)

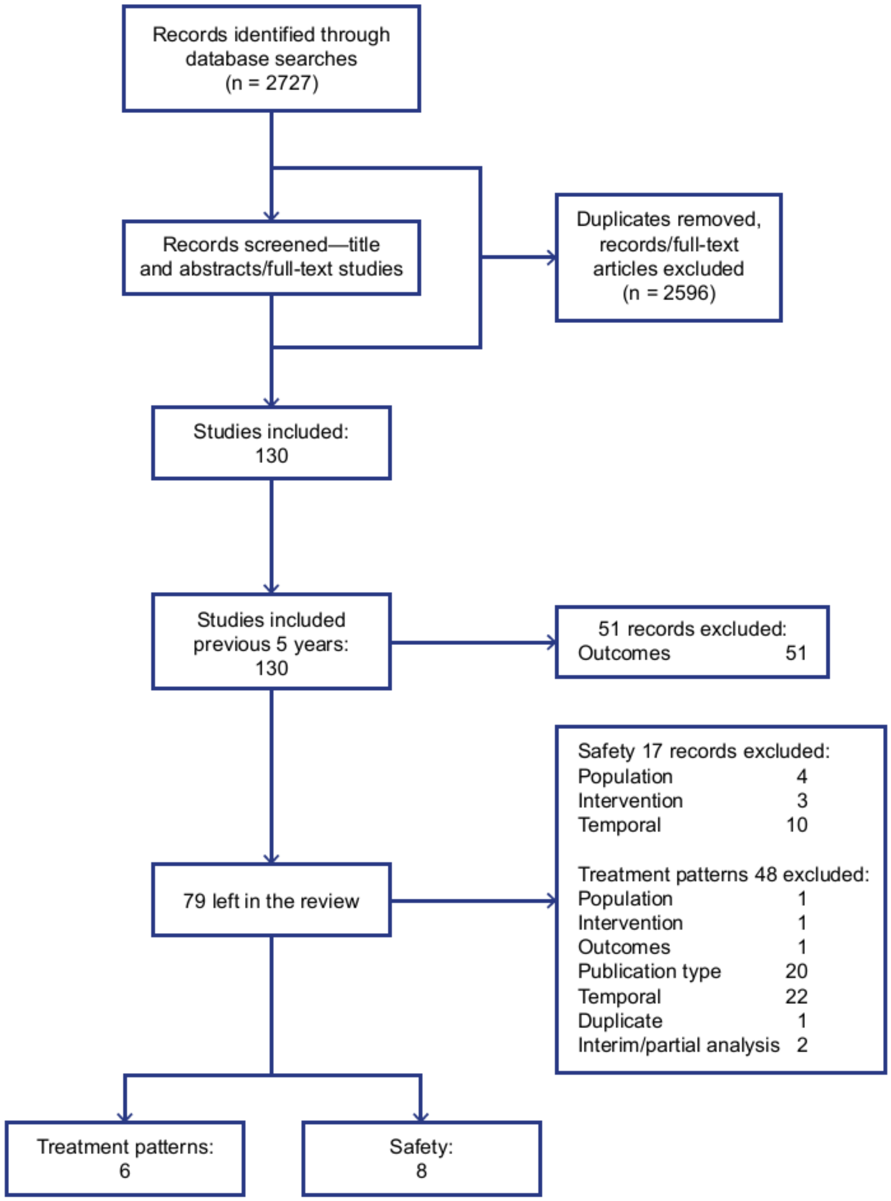

Includes safety data from two studies (three records) from the interventional systematic literature review.

SUPPLEMENTAL FIGURE S3

PRISMA flow of studies included in the systematic literature review of the interventional studies in patients with mCRPC (updated SLR)

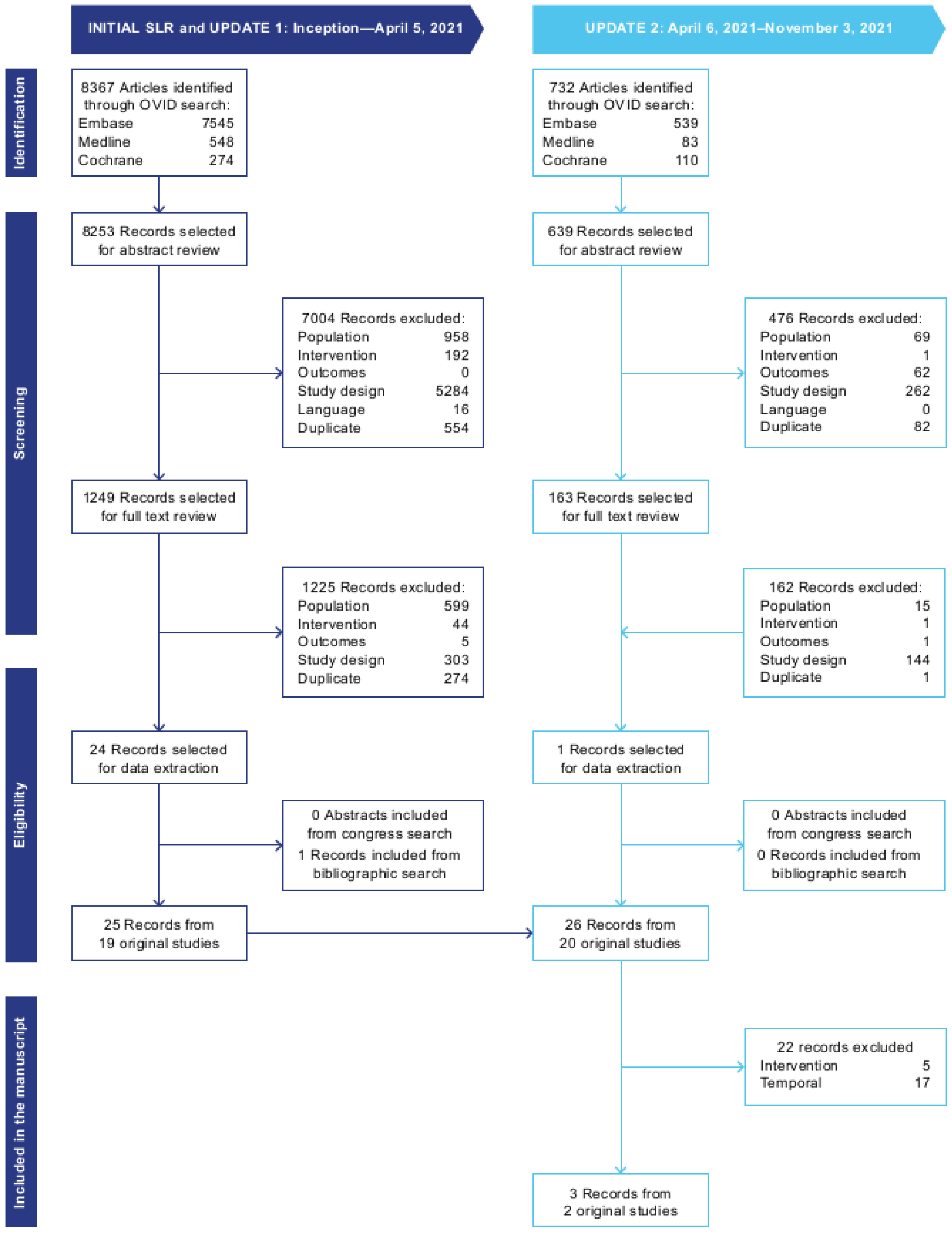

mCRPC: metastatic castration-resistant prostate cancer; PRISMA, Preferred Reporting Items for Systematic Reviews and Meta-Analyses; SLR: systematic literature review.

SUPPLEMENTAL FIGURE S4

PRISMA flow of studies included in the systematic literature review of the health-related quality of life studies in patients with mCRPC (updated SLR)

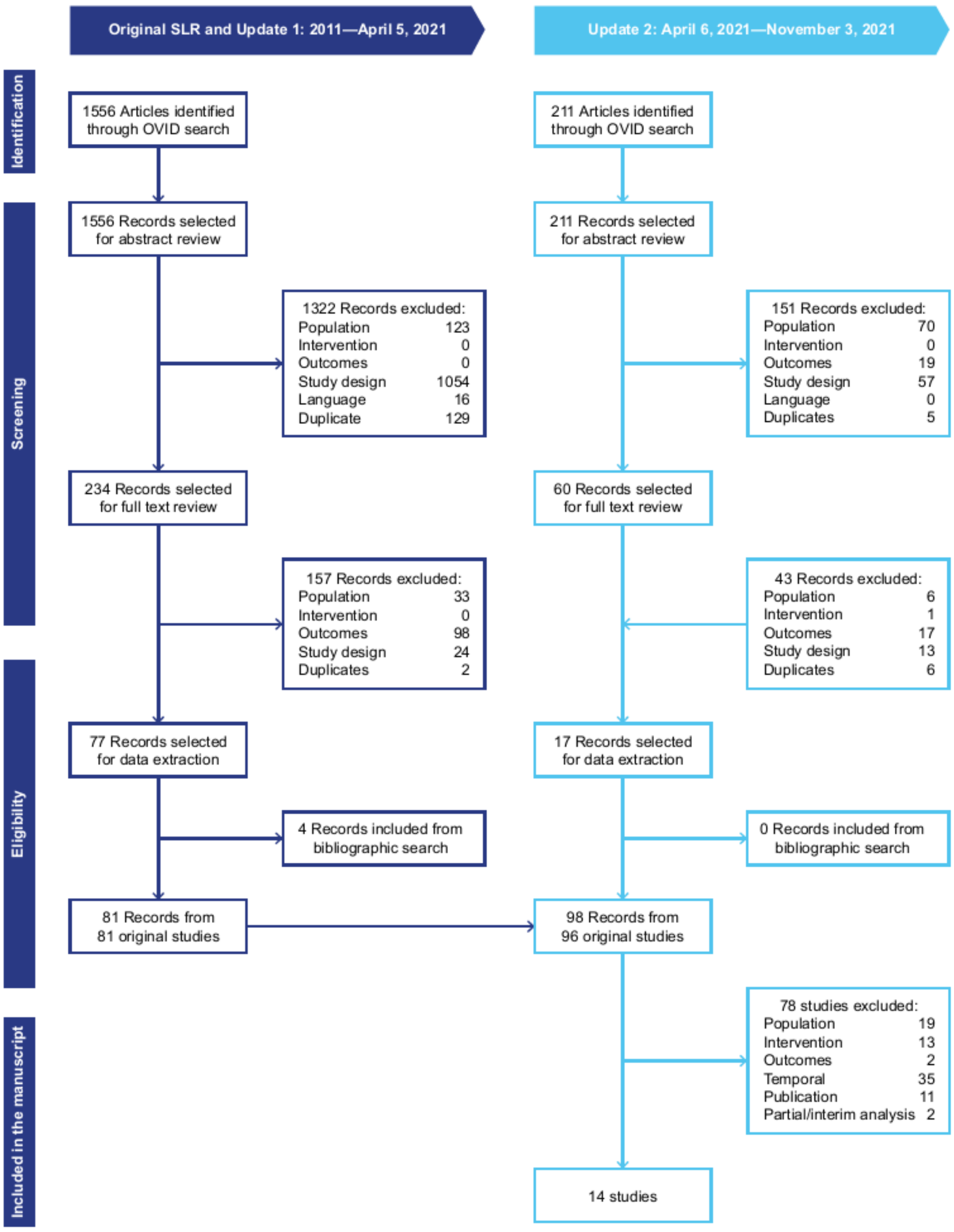

mCRPC, metastatic castration-resistant prostate cancer; PRISMA, Preferred Reporting Items for Systematic Reviews and Meta-Analyses; SLR, systematic literature review.

SUPPLEMENTAL FIGURE S5

PRISMA flow of studies included in the systematic literature review of economic studies in patients with mCRPC (updated SLR)

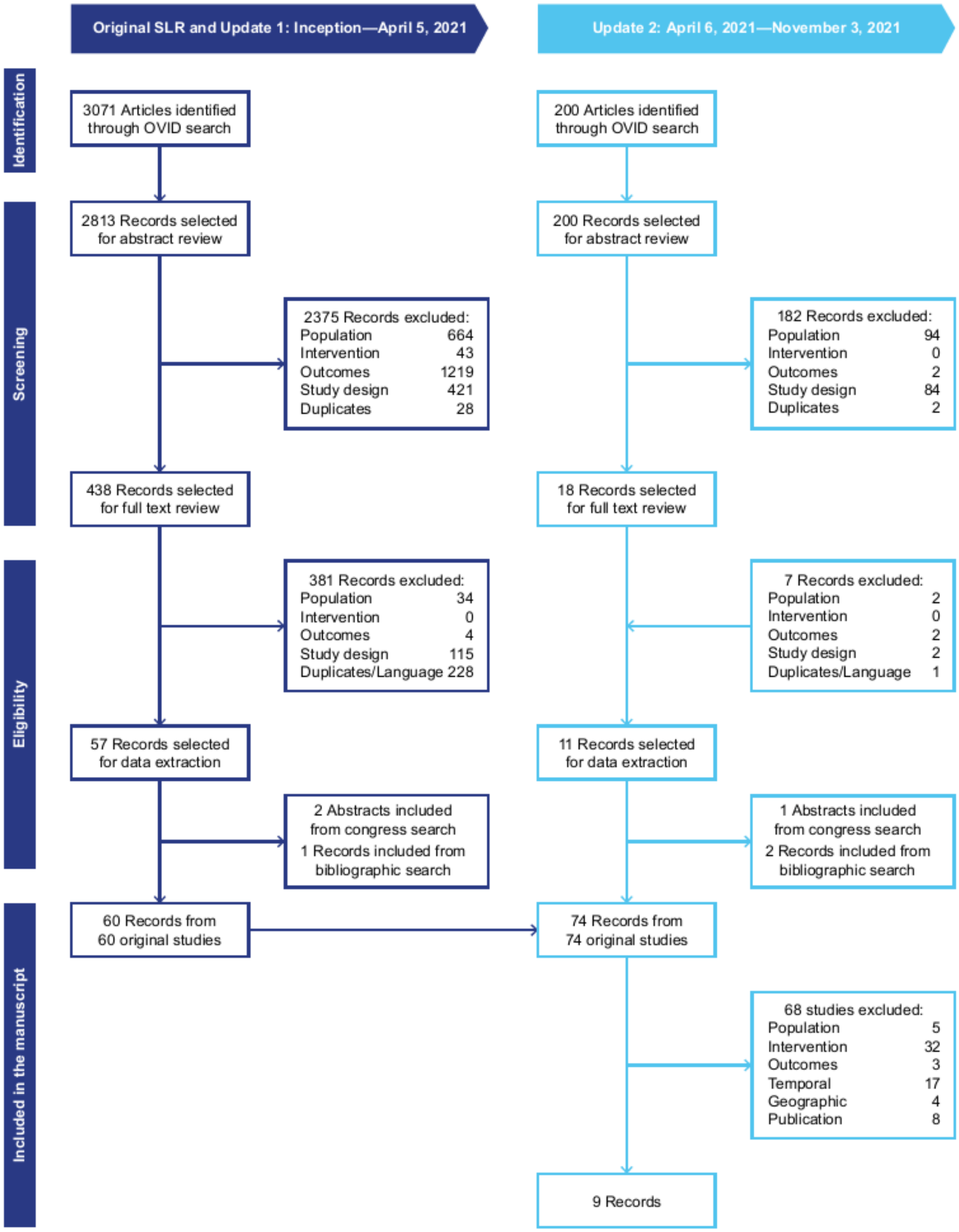

mCRPC, metastatic castration-resistant prostate cancer; PRISMA, Preferred Reporting Items for Systematic Reviews and Meta-Analyses; SLR, systematic literature review.

SUPPLEMENTAL TABLE S1

Treatment landscape review: Embase® search strategy (Original Structured Review)

No.	Search string	Hits
#1	‘metastatic castration resistant prostate cancer’/syn OR ‘metastatic castration resistant prostate cancer’/exp OR mcrpc:ab,ti	5292
#2	‘treatment pattern*’ OR ‘treatment option*’ OR ‘treatment trend*’ OR ‘administration pattern*’ OR ‘prescribing practice*’ OR (treatment NEAR/2 (pattern* OR trend* OR option*)) OR ‘treatment strateg*’ OR ((‘real world’ NEAR/2 (pattern* OR trend* OR practice*)):ab,ti) OR prognos* OR diagnos* OR detect*	9,074,078
#3	‘systemic therap*’ OR chemotherap* OR ‘treatment regimen*’ OR ‘therapeutic agent*’ OR therap*:ab,ti OR treatment*:ab,ti	8,123,052
#4	#1 AND #2 AND #3	2159
#5	#4 AND [2009-2019]/py	2155
#6	#5 AND [english]/lim	2116
#7	#6 AND [animals]/lim NOT ([humans]/lim AND [animals]/lim)	32
#8	#6 AND ([conference review]/lim OR [editorial]/lim OR [erratum]/lim OR [letter]/lim OR [note]/lim OR [review]/lim) NOT ([cochrane review]/lim OR [systematic review]/lim OR [meta analysis]/lim)	220
#9	#6 NOT (#7 OR #8)	1864

SUPPLEMENTAL TABLE S2

Treatment landscape review: MEDLINE® and MEDLINE® in-process search strategy (Original Structured Review)

No.	Search string	Hits
#1	Search ‘metastatic castration resistant prostate cancer’ Sort by: Best Match	2039
#2	Search ‘mcrpc’	1287
#3	Search (advanced OR metastat* OR refract* OR recurren* OR salva* OR ‘late stage’ OR resistan* OR ‘stage iii’ OR ‘stage iv’ OR ‘stage 3’ OR ‘stage 4’ OR ‘metastasis’ OR ‘recurrent disease’)	2,733,109
#4	Search (((castrate OR castration) AND resist*) OR ‘hormone*refractory’ OR ‘androgen*independent’))	14,612
#5	Search (*prostate tumor’ OR ((prostate OR prostatic) AND (neoplasm OR neoplasm* OR cancer OR cancer* OR carcinoma OR carcinoma OR adenocarcinoma OR adenocarcinoma* OR tumour OR tumour* OR tumor OR tumor*)))	171,903
#6	Search (#3 AND #4 AND #5)	11,559
#7	Search (#6 OR #1 OR #2)	11,589
#8	Search (‘treatment pattern’ OR ‘treatment option’ OR ‘treatment trend’ OR ‘administration pattern’ OR ‘prescribing practice’ OR (treatment NEAR/2 (pattern* OR trend* OR option*)) OR ‘treatment strategy’ OR ((‘real world’ NEAR/2 (pattern* OR trend* OR practice*)):ab,ti) OR prognos* OR diagnos* OR detect*)	6,779,617
#9	Search (#8 AND #9)	3807
#10	Search (#10 AND (inprocess[sb] OR pubstatusaheadofprint)))	278

SUPPLEMENTAL TABLE S3

Treatment landscape review: Cochrane search strategy (Original Structured Review)

No.	Query	Results
#1	MeSH descriptor: [Prostatic Neoplasms, Castration-Resistant] explode all trees	189
#2	advanced OR metastat* OR refract* OR recurren* OR salva* OR 'late stage' OR resistan* OR 'stage iii' OR (stage AND iii*) OR 'stage iv' OR 'stage 3' OR 'stage 4' OR 'recurrent disease'	217,317
#3	((castrate OR castration) NEAR/3 resistan*) OR 'hormone*refractory' OR 'hormone refractory' OR 'androgen-independent' OR 'androgen*independent'	2202
#4	'prostate tumor' OR ((prostate OR prostatic) NEAR/2 (neoplasm OR neoplasm* OR cancer OR cancer* OR carcinoma OR carcinoma* OR adenocarcinoma OR adenocarcinoma* OR tumour OR tumour* OR tumor OR tumor*))	13,388
#5	#2 AND #3 AND #4	2122
#6	#1 OR #5	2122
#7	'treatment pattern' OR 'treatment option' OR 'treatment trend' OR 'administration pattern' OR 'prescribing practice' OR (treatment NEAR/2 (pattern* OR trend* OR option*)) OR 'treatment strategy' OR (('real world' NEAR/2 (pattern* OR trend* OR practice*)):ab,ti) OR prognos* OR diagnos* OR detect*	313,605
#8	#6 AND #7	688
#9	#8 with Publication Year from 2009 to 2019, in Trials (Word variations have been searched)	585

SUPPLEMENTAL TABLE S4

Interventional SLR search strategy (Updated Systematic Review)

**INTERVENTIONAL REVIEW**			
**Search conducted**		November 3, 2021	
Databases searched		EBM Reviews - Cochrane Database of Systematic Reviews <2005 to October 27, 2021><br>EBM Reviews - ACP Journal Club <1991 to October 2021><br>EBM Reviews - Database of Abstracts of Reviews of Effects <1st Quarter 2016><br>EBM Reviews - Cochrane Clinical Answers <October 2021><br>EBM Reviews - Cochrane Central Register of Controlled Trials <September 2021><br>EBM Reviews - Cochrane Methodology Register <3rd Quarter 2012><br>EBM Reviews - Health Technology Assessment <4th Quarter 2016><br>EBM Reviews - NHS Economic Evaluation Database <1st Quarter 2016><br>Embase <1974 to 2021 November 02><br>Ovid MEDLINE(R) ALL <1946 to November 02, 2021>	
		**Term**	**Hits**
**Disease: mCRPC**	1	‘exp castration resistant prostate cancer/’ OR ‘exp Prostatic Neoplasms, Castration-Resistant/’	21,026
	2	‘mcrpc.ab,ti.’	9015
	3	exp prostate tumor/ OR exp Prostatic Neoplasms/ OR ((prostate OR prostatic) adj2 (neoplasm OR neoplasm$ OR cancer OR cancer$ OR carcinoma OR carcinoma$ OR adenocarcinoma OR adenocarcinoma$ OR tumour OR tumour$ OR tumor OR tumor$)).ab,ti.	476,636
	4	(((castrate OR castration) adj3 resistan$) OR ‘hormone-refractory’ OR ‘hormone refractory’ OR ‘androgen-independent’ OR ‘androgen independent’).ab,ti.	43,926
	5	(advanced OR metastat$ OR refract$ OR recurren$ OR salva$ OR (‘late’ adj2 ‘stage’) OR resistan$ OR ‘stage iii’ OR (stage and iii$) OR ‘stage iv’ OR ‘stage 3’ OR ‘stage 4’).ti,ab. OR ‘(metastasis/’ OR ‘Neoplasm Metastasis/’ OR ‘exp Recurrence/’ OR ‘recurrent disease/)’	6,604,276
	6	3 AND 4 AND 5	39,710
	7	1 AND 5	19,078

	8	2 OR 6 OR 7	42,074
**Intervention**	9	abiraterone/ OR abiraterone acetate/ OR enzalutamide/ OR docetaxel/ OR cabazitaxel/ OR sipuleucel T/ OR bisphosphonic acid derivative/ OR Diphosphonates/ OR denosumab/ OR radium chloride ra 223/	148,144
	10	(Abiraterone OR Enzalutamide OR Docetaxel OR Cabazitaxel OR Sipuleucel$ OR Bisphosphon$ OR Disphosphon$ OR Denosumab OR Radium$).ti,ab.	128,752
	11	'lutetium 177/' OR 'prostate specific membrane antigen lu 177/' OR 'prostate specific membrane antigen/' OR 'olaparib/' OR 'rucaparib/' OR 'dendritic cell vaccine/' OR 'orteronel/' OR 'pembrolizumab/' OR 'cabozantinib/' OR 'apalutamide/' OR 'sunitinib/' OR 'masitinib/' OR 'talazoparib/' OR 'atezolizumab/' OR 'rhenium 188'/ OR granulocyte macrophage colony stimulating factor vaccine/ OR 'dcvax'/ OR dcvac/ OR tasquinimod/ OR ipatasertib/ OR rilimogene galvacirepvec/ OR rilimogene glafolivec/ OR atrasentan/ OR custirsen/ OR galeterone/	83,947
	12	(177lu-psma-617 OR lu177-psma-617 OR LuPSMA OR Lu-PSMA OR psma-617 OR psma OR Lutetium-177 OR 'Lutetium 177' OR Lutetium177 OR 177Lu-DKFZ-PSMA-617 OR 177Lu-PSMA-DKFZ-617 OR Prostate-specific membrane antigen OR Prostate specific membrane antigen OR olaparib OR rucaparib OR hc-1119 OR dcvac$ OR Autologous dendritic cell vaccin$ OR orteronel OR pembrolizumab OR cabozantinib OR apalutamide OR sunitinib OR masitinib OR talazoparib OR atezolizumab OR rhenium$ OR 188-Re OR 188Re OR '188 Re' OR granulocyte macrophage colony stimulating factor vaccin$ OR gvax OR GM CSF vaccin$ OR GM CSF gene vaccin$ OR GMCSF vaccin$ OR GMCSF gene vaccin$ OR dcvax$ OR tasquinimod OR ipatasertib OR rilimogene$ OR PROSTVAC$ OR rilimogene galvacirepvec OR rilimogene glafolivec OR atrasentan OR custirsen OR galeterone).ti,ab.	77,034
	13	or/9-12	301,631
**Interventional studies**	14	exp Randomized Controlled Trial/ OR exp Random Allocation/ OR exp randomization/	1,414,112
	15	exp Placebos/	436,236

https://www.nice.org.uk/guidance/ng50/documents/search-strategies	16	exp Double-Blind Method/ OR exp Single-Blind Method/	595,909
	17	exp clinical trial/ OR exp clinical trial, phase ii/ OR exp clinical trial, phase iii/ OR exp controlled clinical trial/	2,559,274
	18	exp controlled clinical trials as topic/ OR exp Randomized Controlled Trials as Topic/ OR exp clinical trials as topic/	781,210
	19	exp Multicenter Study/	612,218
	20	exp Randomized Controlled Trial/ OR exp Random Allocation/ OR exp randomization/	1,414,112
	21	exp placebo/	373,103
	22	exp double blind procedure/ OR exp single blind procedure/ OR exp crossover procedure/	274,647
	23	exp clinical trial/ OR exp phase 2 clinical trial/ OR exp phase 3 clinical trial/ OR exp controlled clinical trial/	2,559,274
	24	exp 'controlled clinical trial (topic)'/ OR exp 'clinical trial (topic)'/ OR exp 'randomized controlled trial (topic)'/	372,547
	25	exp multicenter Study/	612,218
	26	randomized controlled trial.pt.	1,083,897
	27	controlled clinical trial.pt.	186,992
	28	random$.ti,ab,kw.	4,168,600
	29	blind$.ti,ab,kw.	1,155,773
	30	(placebo$ OR assign* OR allocat* OR volunteer*).ti,ab,kw.	2,603,661
	31	(parallel$ OR fact OR ial$ OR crossover* OR cross over*).ti,ab,kw.	1,169,186
	32	trial.ti.	959,354
	33	('phase 3' OR 'phase 2' OR 'phase III' OR 'phase II').af.	594,131
	34	((single OR double OR triple) adj3 (blind* OR mask* OR dummy)).af.	1,010,464
	35	('double-blind' OR 'double-blinded').af.	846,745
	36	(open label OR open-label).af.	219,341
	37	('single arm' OR 'single-arm' OR 'single group' OR 'single-group').ti,ab.	44,262
**SLR** https://www.nice.org.uk/guidance/ng50/doc	38	exp Meta-Analysis/ OR exp Meta-Analysis as Topic/ OR exp 'Systematic Review'/	733,179
	39	exp meta analysis/ OR exp 'meta analysis (topic)'/ OR exp 'systematic review'/	713,811

uments/search<br>-strategies	40	(meta analy* OR meta-analy* OR metanaly* OR metaanaly*).ti,ab.	536,753
	41	((systematic* OR evidence*) adj3 (review* OR overview*)).ti,ab.	672,739
	42	(reference list* OR bibliograph* OR hand search* OR manual search* OR relevant journal*).ab.	118,671
	43	(search strategy OR search criteria OR systematic search OR study selection OR data extraction).ab.	156,980
	44	(search* adj4 literature).ab.	184,592
	45	(medline OR pubmed OR cochrane OR embase OR psychlit OR psyclit OR psychinfo OR psycinfo OR cinahl OR science citation index OR bids OR cancerlit).ab.	674,227
	46	cochrane.jw.	54,226
	47	((multiple treatment* OR indirect OR mixed) adj2 comparison*).ti,ab.	10,479
	48	or/14-47	9,718,818
**Interventional studies or SLR in treated patients with mCRPC**	**49**	**8 AND 13 AND 48**	**12,035**
**Irrelevant Study Design**	50	(addresses OR bibliography OR biography OR case report OR comment OR congresses OR consensus development conference OR duplicate publication OR editorial OR guideline OR *in vitro* OR interview OR lectures OR letter OR monograph OR news OR 'newspaper article' OR practice guideline OR 'review literature' OR 'review of reported cases' OR review, academic OR review, multicase OR review, tutorial OR twin study).pt.	4,396,415
	51	(animals/ not (humans/ and animals/)) OR (animal/ not (human/ and animal/))	6,050,706
	52	case report/ OR case reports/	4,894,359
	53	50 OR 51 OR 52	14,725,834
	54	49 not 53	11,594
**Limit**	55	limit 54 to English language	10,826
	56	limit 55 to human	10,213

	57	limit 56 to yr=‘2021 -Current’	732
**Final Interventional studies or SLR in treated patients with mCRPC**	**58**	**remove duplicates from 57**	**639**

ACP: American College of Physicians; EBM: Evidence-Based Medicine; mCRPC: metastatic castration-resistant prostate cancer; NHS: National Health Service; SLR: systematic literature review.

SUPPLEMENTAL TABLE S5

Interventional SLR search strategy—Medline® (Updated Systematic Review)

**Search conducted**	October 29, 2021		
**Databases searched**	Ovid MEDLINE(R) ALL <1946 to October 28, 2021>		
		**Term**	**Hits**
**Disease: mCRPC**	1	exp castration resistant prostate cancer/ OR exp Prostatic Neoplasms, Castration-Resistant/	4809
	2	mcrpc.ab,ti.	2096
	3	exp prostate tumor/ OR exp Prostatic Neoplasms/ OR ((prostate OR prostatic) adj2 (neoplasm OR neoplasm$ OR cancer OR cancer$ OR carcinoma OR carcinoma$ OR adenocarcinoma OR adenocarcinoma$ OR tumour OR tumour$ OR tumor OR tumor$)).ab,ti.	175,614
	4	(((castrate OR castration) adj3 resistan$) OR ‘hormone-refractory’ OR ‘hormone refractory’ OR ‘androgen-independent’ OR ‘androgen independent’).ab,ti.	15,275
	5	(advanced OR metastat$ OR refract$ OR recurren$ OR salva$ OR (‘late’ adj2 ‘stage’) OR resistan$ OR ‘stage iii‘ OR (stage and iii$) OR ‘stage iv’ OR ‘stage 3’ OR ‘stage 4’).ti,ab. OR (metastasis/ OR Neoplasm Metastasis/ OR exp Recurrence/ OR recurrent disease/)	2,685,168
	6	3 AND 4 AND 5	13,404
	7	1 AND 5	4398
	8	2 OR 6 OR 7	13,782
**Intervention**	9	abiraterone/ OR abiraterone acetate/ OR enzalutamide/ OR docetaxel/ OR cabazitaxel/ OR sipuleucel T/ OR bisphosphonic acid derivative/ OR Diphosphonates/ OR denosumab/ OR radium chloride ra 223/	29,689
	10	(Abiraterone OR Enzalutamide OR Docetaxel OR Cabazitaxel OR Sipuleucel$ OR Bisphosphon$ OR Disphosphon$ OR Denosumab OR Radium$).ti,ab.	44,621

	11	lutetium 177/ OR prostate specific membrane antigen lu 177/ OR prostate specific membrane antigen/ OR olaparib/ OR rucaparib/ OR dendritic cell vaccine/ OR orteronel/ OR pembrolizumab/ OR cabozantinib/ OR apalutamide/ OR sunitinib/ OR masitinib/ OR talazoparib/ OR atezolizumab/ OR ‘rhenium 188‘/ OR granulocyte macrophage colony stimulating factor vaccine/ OR ‘dcvax‘/ OR dcvac/ OR tasquinimod/ OR ipatasertib/ OR rilimogene galvacirepvec/ OR rilimogene glafolivec/ OR atrasentan/ OR custirsen/ OR galeterone/	4144
	12	(177lu-psma-617 OR lu177-psma-617 OR LuPSMA OR Lu-PSMA OR psma-617 OR psma OR Lutetium-177 OR ‘Lutetium 177’ OR Lutetium177 OR 177Lu-DKFZ-PSMA-617 OR 177Lu-PSMA-DKFZ-617 OR Prostate-specific membrane antigen OR Prostate specific membrane antigen OR olaparib OR rucaparib OR hc-1119 OR dcvac$ OR Autologous dendritic cell vaccin$ OR orteronel OR pembrolizumab OR cabozantinib OR apalutamide OR sunitinib OR masitinib OR talazoparib OR atezolizumab OR rhenium$ OR 188-Re OR 188Re OR ‘188 Re‘ OR granulocyte macrophage colony stimulating factor vaccin$ OR gvax OR GM CSF vaccin$ OR GM CSF gene vaccin$ OR GMCSF vaccin$ OR GMCSF gene vaccin$ OR dcvax$ OR tasquinimod OR ipatasertib OR rilimogene$ OR PROSTVAC$ OR rilimogene galvacirepvec OR rilimogene glafolivec OR atrasentan OR custirsen OR galeterone).ti,ab.	23,889
	13	or/9-12	76,868
**Interventional studies** https://www.nice.org.uk/guidance/ng50/documents/search-strategies	14	exp Randomized Controlled Trial/ OR exp Random Allocation/ OR exp randomization/	639,063
	15	exp Placebos/	38,708
	16	exp Double-Blind Method/ OR exp Single-Blind Method/	198,231
	17	exp clinical trial/ OR exp clinical trial, phase ii/ OR exp clinical trial, phase iii/ OR exp controlled clinical trial/	916,490
	18	exp controlled clinical trials as topic/ OR exp Randomized Controlled Trials as Topic/ OR exp clinical trials as topic/	365,985
	19	exp Multicenter Study/	307,574
	20	exp Randomized Controlled Trial/ OR exp Random Allocation/ OR exp randomization/	639,063
	21	exp placebo/	0

	22	exp double blind procedure/ OR exp single blind procedure/ OR exp crossover procedure/	0
	23	exp clinical trial/ OR exp phase 2 clinical trial/ OR exp phase 3 clinical trial/ OR exp controlled clinical trial/	916,490
	24	exp ‘controlled clinical trial (topic)’/ OR exp ‘clinical trial (topic)’/ OR exp ‘randomized controlled trial (topic)’/	0
	25	exp multicenter Study/	307,574
	26	randomized controlled trial.pt.	549,431
	27	controlled clinical trial.pt.	94,513
	28	random$.ti,ab,kw.	1,267,648
	29	blind$.ti,ab,kw.	321,081
	30	(placebo$ OR assign* OR allocat* OR volunteer*).ti,ab,kw.	854,640
	31	(parallel$ OR factorial$ OR crossover* OR cross over*).ti,ab,kw.	453,033
	32	trial.ti.	250,471
	33	(‘phase 3’ OR ‘phase 2’ OR ‘phase III’ OR ‘phase II’).af.	146,558
	34	((single OR double OR triple) adj3 (blind* OR mask* OR dummy)).af.	253,986
	35	(‘double-blind’ OR ‘double-blinded’).af.	212,563
	36	(open label OR open-label).af.	48,151
	37	(‘single arm’ OR ‘single-arm’ OR ‘single group’ OR ‘single-group’).ti,ab.	13,761
**SLR** https://www.nice.org.uk/guidance/ng50/documents/search-strategies	38	exp Meta-Analysis/ OR exp Meta-Analysis as Topic/ OR exp ‘Systematic Review’/	265,015
	39	exp meta analysis/ OR exp ‘meta analysis (topic)’/ OR exp ‘systematic review’/	245,929
	40	(meta analy* OR meta-analy* OR metanaly* OR metaanaly*).ti,ab.	216,880
	41	((systematic* OR evidence*) adj3 (review* OR overview*)).ti,ab.	286,519
	42	(reference list* OR bibliograph* OR hand search* OR manual search* OR relevant journal*).ab.	48,642
	43	(search strategy OR search criteria OR systematic search OR study selection OR data extraction).ab.	66,597
	44	(search* adj4 literature).ab.	79,725
	45	(medline OR pubmed OR cochrane OR embase OR psychlit OR psyclit OR psychinfo OR psycinfo OR cinahl OR science citation index OR bids OR cancerlit).ab.	287,839
	46	cochrane.jw.	15,696
	47	((multiple treatment* OR indirect OR mixed) adj2 comparison*).ti,ab.	3288

	48	or/14-47	3,462,293
**Interventional studies OR SLR in treated patients with mCRPC**	**49**	**8 AND 13 AND 48**	**2458**
**Irrelevant Study Design**	50	(addresses OR bibliography OR biography OR case report OR comment OR congresses OR consensus development conference OR duplicate publication OR editorial OR guideline OR *in vitro* OR interview OR lectures OR letter OR monograph OR news OR 'newspaper article' OR practice guideline OR 'review literature' OR 'review of reported cases' OR review, academic OR review, multicase OR review, tutorial OR twin study).pt.	2,483,480
	51	(animals/ not (humans/ and animals/)) OR (animal/ not (human/ and animal/))	4,876,158
	52	case report/ OR case reports/	2,222,460
	53	50 OR 51 OR 52	9,235,998
	54	49 not 53	2321
**Limit**	55	limit 54 to English language	2203
	56	limit 55 to human	1912
**Final Interventional studies OR SLRs in treated patients with mCRPC (MEDLINE)**	**57**	**limit 56 to yr='2021 -Current'**	**83**

mCRPC: metastatic castration-resistant prostate cancer; SLR: systematic literature review.

SUPPLEMENTAL TABLE S6

Interventional SLR search strategy – Embase® (updated systematic review)

**Search conducted**	November 3, 2021		
**Databases searched**	Embase <1974 to 2021 November 02>		
		**Term**	**Hits**
	1	exp castration resistant prostate cancer/ OR exp Prostatic Neoplasms, Castration-Resistant/	15,931
	2	mcrpc.ab,ti.	5845
	3	exp prostate tumor/ OR exp Prostatic Neoplasms/ OR ((prostate OR prostatic) adj2 (neoplasm OR neoplasm$ OR cancer OR cancer$ OR carcinoma OR carcinoma$ OR adenocarcinoma OR adenocarcinoma$ OR tumour OR tumour$ OR tumor OR tumor$)).ab,ti.	285,313
**Disease: mCRPC**	4	(((castrate OR castration) adj3 resistan$) OR ‘hormone-refractory’ OR ‘hormone refractory’ OR ‘androgen-independent’ OR ‘androgen independent’).ab,ti.	25,720
	5	(advanced OR metastat$ OR refract$ OR recurren$ OR salva$ OR (‘late’ adj2 ‘stage’) OR resistan$ OR ‘stage iii’ OR (stage and iii$) OR ‘stage iv’ OR ‘stage 3’ or ‘stage 4’).ti,ab. OR (metastasis/ OR Neoplasm Metastasis/ OR exp Recurrence/ OR recurrent disease/)	3,674,027
	6	3 AND 4 AND 5	23,478
	7	1 AND 5	14,402
	8	2 OR 6 OR 7	25,354
**Intervention**	9	abiraterone/ OR abiraterone acetate/ OR enzalutamide/ OR docetaxel/ OR cabazitaxel/ OR sipuleucel T/ OR bisphosphonic acid derivative/ OR Diphosphonates/ OR denosumab/ OR radium chloride ra 223/	114,510
	10	(Abiraterone OR Enzalutamide OR Docetaxel OR Cabazitaxel OR Sipuleucel$ OR Bisphosphon$ OR Disphosphon$ OR Denosumab OR Radium$).ti,ab.	71,869

	11	‘lutetium 177/ OR prostate specific membrane antigen lu 177/ OR prostate specific membrane antigen/ OR olaparib/ OR rucaparib/ OR dendritic cell vaccine/ OR orteronel/ OR pembrolizumab/ OR cabozantinib/ OR apalutamide/ OR sunitinib/ OR masitinib/ OR talazoparib/ OR atezolizumab/ OR ‘rhenium 188‘/ OR granulocyte macrophage colony stimulating factor vaccine/ OR ‘dcvax‘/ OR dcvac/ OR tasquinimod/ OR ipatasertib/ OR rilimogene galvacirepvec/ OR rilimogene glafolivec/ OR atrasentan/ OR custirsen/ OR galeterone/	78,776
	12	(177lu-psma-617 OR lu177-psma-617 OR LuPSMA OR Lu-PSMA OR psma-617 OR psma OR Lutetium-177 OR ‘Lutetium 177’ OR Lutetium177 OR 177Lu-DKFZ-PSMA-617 OR 177Lu-PSMA-DKFZ-617 OR Prostate-specific membrane antigen OR Prostate specific membrane antigen OR olaparib OR rucaparib OR hc-1119 OR dcvac$ OR Autologous dendritic cell vaccin$ OR orteronel OR pembrolizumab OR cabozantinib OR apalutamide OR sunitinib OR masitinib OR talazoparib OR atezolizumab OR rhenium$ OR 188-Re OR 188Re OR ‘188 Re‘ OR granulocyte macrophage colony stimulating factor vaccin$ OR gvax OR GM CSF vaccin$ OR GM CSF gene vaccin$ OR GMCSF vaccin$ OR GMCSF gene vaccin$ OR dcvax$ OR tasquinimod OR ipatasertib OR rilimogene$ OR PROSTVAC$ OR rilimogene galvacirepvec OR rilimogene glafolivec OR atrasentan OR custirsen OR galeterone).ti,ab	46,634
	13	or/9-12	205,711
**Interventional studies** https://www.nice.org.uk/guidance/ng50/documents/search-strategies	14	exp Randomized Controlled Trial/ OR exp Random Allocation/ OR exp randomization/	754,207
	15	exp Placebos/	373,103
	16	exp Double-Blind Method/ OR exp Single-Blind Method/	231,274
	17	exp clinical trial/ OR exp clinical trial, phase ii/ OR exp clinical trial, phase iii/ OR exp controlled clinical trial/	1,642,620
	18	exp controlled clinical trials as topic/ OR exp Randomized Controlled Trials as Topic/ OR exp clinical trials as topic/	372,547
	19	exp Multicenter Study/	304,633
	20	exp Randomized Controlled Trial/ OR exp Random Allocation/ OR exp randomization/	754,207
	21	exp placebo/	373,103

	22	exp double blind procedure/ OR exp single blind procedure/ OR exp crossover procedure/	274,647
	23	exp clinical trial/ OR exp phase 2 clinical trial/ OR exp phase 3 clinical trial/ OR exp controlled clinical trial/	1,642,620
	24	exp ‘controlled clinical trial (topic)’/ OR exp ‘clinical trial (topic)’/ OR exp ‘randomized controlled trial (topic)’/	372,547
	25	exp multicenter Study/	304,633
	26	randomized controlled trial.pt.	0
	27	controlled clinical trial.pt.	0
	28	random$.ti,ab,kw.	1,725,715
	29	blind$.ti,ab,kw.	456,821
	30	(placebo$ OR assign* OR allocat* OR volunteer*).ti,ab,kw.	1,134,406
	31	(parallel$ OR factorial$ OR crossover* OR cross over*).ti,ab,kw.	542,494
	32	trial.ti.	342,368
	33	(‘phase 3’ OR ‘phase 2’ OR ‘phase III’ OR ‘phase II’).af.	305,490
	34	((single OR double OR triple) adj3 (blind* OR mask* OR dummy)).af.	332,745
	35	(‘double-blind‘ OR ‘double-blinded‘).af.	275,275
	36	(open label OR open-label).af.	92,054
	37	(‘single arm’ OR ‘single-arm’ OR ‘single group’ OR ‘single-group’).ti,ab.	25,994
**SLR** https://www.nice.org.uk/guidance/ng50/documents/search-strategies	38	exp Meta-Analysis/ OR exp Meta-Analysis as Topic/ OR exp ‘Systematic Review’/	467,859
	39	exp meta analysis/ OR exp ‘meta analysis (topic)’/ OR exp ‘systematic review’/	467,859
	40	(meta analy* OR meta-analy* OR metanaly* OR metaanaly*).ti,ab.	280,046
	41	((systematic* OR evidence*) adj3 (review* OR overview*)).ti,ab.	348,284
	42	(reference list* OR bibliograph* OR hand search* OR manual search* OR relevant journal*).ab.	59,539
	43	(search strategy OR search criteria OR systematic search OR study selection OR data extraction).ab.	79,951
	44	(search* adj4 literature).ab.	100,842
	45	(medline OR pubmed OR cochrane OR embase OR psychlit OR psyclit OR psychinfo OR psycinfo OR cinahl OR science citation index OR bids OR cancerlit).ab.	353,945
	46	cochrane.jw.	23,290
	47	((multiple treatment* OR indirect OR mixed) adj2 comparison*).ti,ab.	5987

	48	or/14-47	4,668,717
**Interventional studies OR SLRs in treated patients with mCRPC**	**49**	**8 AND 13 AND 48**	**7494**
**Irrelevant Study Design**	50	(addresses OR bibliography OR biography OR case report OR comment OR congresses OR consensus development conference OR duplicate publication OR editorial OR guideline OR *in vitro* OR interview OR lectures OR letter OR monograph OR news OR 'newspaper article' OR practice guideline OR 'review literature' OR 'review of reported cases' OR review, academic OR review, multicase OR review, tutorial OR twin study).pt.	1,903,580
	51	(animals/ not (humans/ and animals/)) OR (animal/ not (human/ and animal/))	1,163,841
	52	case report/ OR case reports/	2,671,895
	53	50 OR 51 OR 52	5,469,896
	54	49 not 53	7198
**Limit**	55	limit 54 to English language	7029
	56	limit 55 to human	6709
**Final Interventional studies OR SLR in treated patients with mCRPC (Embase)**	**57**	**limit 56 to yr='2021 -Current'**	**539**

mCRPC: metastatic castration-resistant prostate cancer; SLR: systematic literature review.

SUPPLEMENTAL TABLE S7

Interventional SLR search strategy—Cochrane (updated systematic review)

**Search conducted**	November 3, 2021		
**Databases searched**	EBM Reviews - Cochrane Database of Systematic Reviews <2005 to October 27, 2021><br>EBM Reviews - ACP Journal Club <1991 to October 2021><br>EBM Reviews - Database of Abstracts of Reviews of Effects <1st Quarter 2016><br>EBM Reviews - Cochrane Clinical Answers <October 2021><br>EBM Reviews - Cochrane Central Register of Controlled Trials <September 2021><br>EBM Reviews - Cochrane Methodology Register <3rd Quarter 2012><br>EBM Reviews - Health Technology Assessment <4th Quarter 2016><br>EBM Reviews - NHS Economic Evaluation Database <1st Quarter 2016>		
		**Term**	**Hits**
**Disease: mCRPC**	1	exp castration resistant prostate cancer/ OR exp Prostatic Neoplasms, Castration-Resistant/	286
	2	mcrpc.ab,ti.	1074
	3	exp prostate tumor/ OR exp Prostatic Neoplasms/ OR ((prostate OR prostatic) adj2 (neoplasm OR neoplasm$ OR cancer OR cancer$ OR carcinoma OR carcinoma$ OR adenocarcinoma OR adenocarcinoma$ OR tumour OR tumour$ OR tumor OR tumor$)).ab,ti.	15,709
	4	(((castrate OR castration) adj3 resistan$) OR ‘hormone-refractory‘ OR ‘hormone refractory’ OR ‘androgen-independent’ OR ‘androgen independent’).ab,ti.	2931
	5	(advanced OR metastat$ OR refract$ OR recurren$ OR salva$ OR (‘late’ adj2 ‘stage’) OR resistan$ OR ‘stage iii’ OR (stage and iii$) OR ‘stage iv’ OR ‘stage 3’ OR ‘stage 4’).ti,ab. OR (metastasis/ OR Neoplasm Metastasis/ OR exp Recurrence/ OR recurrent disease/)	245,081
	6	3 AND 4 AND 5	2828
	7	1 AND 5	278
	8	2 OR 6 OR 7	2938
**Intervention**	9	abiraterone/ OR abiraterone acetate/ OR enzalutamide/ OR docetaxel/ OR cabazitaxel/ OR sipuleucel T/ OR bisphosphonic acid derivative/ OR Diphosphonates/ OR denosumab/ OR radium chloride ra 223/	3945

	10	(Abiraterone OR Enzalutamide OR Docetaxel OR Cabazitaxel OR Sipuleucel$ OR Bisphosphon$ OR Disphosphon$ OR Denosumab OR Radium$).ti,ab.	12,262
	11	lutetium 177/ OR prostate specific membrane antigen lu 177/ OR prostate specific membrane antigen/ OR olaparib/ OR rucaparib/ OR dendritic cell vaccine/ OR orteronel/ OR pembrolizumab/ OR cabozantinib/ OR apalutamide/ OR sunitinib/ OR masitinib/ OR talazoparib/ OR atezolizumab/ OR 'rhenium 188'/ OR granulocyte macrophage colony stimulating factor vaccine/ OR 'dcvax'/ OR dcvac/ OR tasquinimod/ OR ipatasertib/ OR rilimogene galvacirepvec/ OR rilimogene glafolivec/ OR atrasentan/ OR custirsen/ OR galeterone/	1027
	12	(177lu-psma-617 OR lu177-psma-617 OR LuPSMA OR Lu-PSMA OR psma-617 OR psma OR Lutetium-177 OR 'Lutetium 177' OR Lutetium177 OR 177Lu-DKFZ-PSMA-617 OR 177Lu-PSMA-DKFZ-617 OR Prostate-specific membrane antigen OR Prostate specific membrane antigen OR olaparib OR rucaparib OR hc-1119 OR dcvac$ OR Autologous dendritic cell vaccin$ OR orteronel OR pembrolizumab OR cabozantinib OR apalutamide OR sunitinib OR masitinib OR talazoparib OR atezolizumab OR rhenium$ OR 188-Re OR 188Re OR '188 Re' OR granulocyte macrophage colony stimulating factor vaccin$ OR gvax OR GM CSF vaccin$ OR GM CSF gene vaccin$ OR GMCSF vaccin$ OR GMCSF gene vaccin$ OR dcvax$ OR tasquinimod OR ipatasertib OR rilimogene$ OR PROSTVAC$ OR rilimogene galvacirepvec OR rilimogene glafolivec OR atrasentan OR custirsen OR galeterone).ti,ab.	6511
	13	or/9-12	19,052
**Interventional studies** https://www.nice.org.uk/guidance/ng50/documents/search-strategies	14	exp Randomized Controlled Trial/ OR exp Random Allocation/ OR exp randomization/	20,842
	15	exp Placebos/	24,425
	16	exp Double-Blind Method/ OR exp Single-Blind Method/	166,404
	17	exp clinical trial/ OR exp clinical trial, phase ii/ OR exp clinical trial, phase iii/ OR exp controlled clinical trial/	164
	18	exp controlled clinical trials as topic/ OR exp Randomized Controlled Trials as Topic/ OR exp clinical trials as topic/	42,678
	19	exp Multicenter Study/	11

	20	exp Randomized Controlled Trial/ OR exp Random Allocation/ OR exp randomization/	20,842
	21	exp placebo/	0
	22	exp double blind procedure/ OR exp single blind procedure/ OR exp crossover procedure/	0
	23	exp clinical trial/ OR exp phase 2 clinical trial/ OR exp phase 3 clinical trial/ OR exp controlled clinical trial/	164
	24	exp ‘controlled clinical trial (topic)’/ OR exp ‘clinical trial (topic)’/ OR exp ‘randomized controlled trial (topic)’/	0
	25	exp multicenter Study/	11
	26	randomized controlled trial.pt.	534,466
	27	controlled clinical trial.pt.	92,479
	28	random$.ti,ab,kw.	1,175,237
	29	blind$.ti,ab,kw.	377,871
	30	(placebo$ OR assign* OR allocat* OR volunteer*).ti,ab,kw.	614,615
	31	(parallel$ OR factorial$ OR crossover* OR cross over*).ti,ab,kw.	173,659
	32	trial.ti.	366,515
	33	(‘phase 3‘ OR ‘phase 2‘ OR ‘phase III‘ OR ‘phase II‘).af.	142,083
	34	((single OR double OR triple) adj3 (blind* OR mask* OR dummy)).af.	423,733
	35	(‘double-blind‘ OR ‘double-blinded‘).af.	358,907
	36	(open label OR open-label).af.	79,136
	37	(‘single arm’ OR ‘single-arm’ OR ‘single group’ OR ‘single-group’).ti,ab.	4507
**SLR** https://www.nice.org.uk/guidance/ng50/documents/search-strategies	38	exp Meta-Analysis/ OR exp Meta-Analysis as Topic/ OR exp ‘Systematic Review’/	305
	39	exp meta analysis/ OR exp ‘meta analysis (topic)’/ OR exp ‘systematic review’/	23
	40	(meta analy* OR meta-analy* OR metanaly* OR metaanaly*).ti,ab.	39,827
	41	((systematic* OR evidence*) adj3 (review* OR overview*)).ti,ab.	37,936
	42	(reference list* OR bibliograph* OR hand search* OR manual search* OR relevant journal*).ab.	10,490
	43	(search strategy OR search criteria OR systematic search OR study selection OR data extraction).ab.	10,432
	44	(search* adj4 literature).ab.	4025

	45	(medline OR pubmed OR cochrane OR embase OR psychlit OR psyclit OR psychinfo OR psycinfo OR cinahl OR science citation index OR bids OR cancerlit).ab.	32,443
	46	cochrane.jw.	15,240
	47	((multiple treatment* OR indirect OR mixed) adj2 comparison*).ti,ab.	1204
	48	or/14-47	1,587,808
**Interventional studies OR SLRs in treated patients with mCRPC**	**49**	**8 AND 13 AND 48**	**2083**
**Irrelevant Study Design**	50	(addresses OR bibliography OR biography OR case report OR comment OR congresses OR consensus development conference OR duplicate publication OR editorial OR guideline OR *in vitro* OR interview OR lectures OR letter OR monograph OR news OR 'newspaper article' OR practice guideline OR 'review literature' OR 'review of reported cases' OR review, academic OR review, multicase OR review, tutorial OR twin study).pt.	9355
	51	(animals/ not (humans/ and animals/)) OR (animal/ not (human/ and animal/))	10,707
	52	case report/ OR case reports/	4
	53	50 OR 51 OR 52	19,940
	54	49 not 53	2075
**Limit**	55	limit 54 to English language	1594
	56	limit 55 to human	1592
**Final Interventional studies OR SLRs in treated patients with mCRPC (Cochrane)**	**57**	**limit 56 to yr='2021 -Current'**	**110**

ACP, American College of Physicians; EBM, Evidence-Based Medicine; mCRPC, metastatic castration-resistant prostate cancer; NHS, National Health Service; SLR, systematic literature review.

SUPPLEMENTAL TABLE S8

Health-Related Quality of Life SLR search strategy (updated systematic review)

**HRQOL Review**			
**Search conducted**	November 3, 2021		
**Databases searched**	EBM Reviews - Cochrane Database of Systematic Reviews <2005 to October 27, 2021><br>EBM Reviews - ACP Journal Club <1991 to October 2021><br>EBM Reviews - Database of Abstracts of Reviews of Effects <1st Quarter 2016><br>EBM Reviews - Cochrane Clinical Answers <October 2021><br>EBM Reviews - Cochrane Central Register of Controlled Trials <September 2021><br>EBM Reviews - Cochrane Methodology Register <3rd Quarter 2012><br>EBM Reviews - Health Technology Assessment <4th Quarter 2016><br>EBM Reviews - NHS Economic Evaluation Database <1st Quarter 2016><br>EconLit <1886 to October 21, 2021><br>Embase <1974 to 2021 November 02><br>Ovid MEDLINE(R) ALL <1946 to November 02, 2021>		
		**Term**	**Hits**
**Disease: mCRPC**	1	exp castration resistant prostate cancer/ OR exp Prostatic Neoplasms, Castration-Resistant/	21,026
	2	mcrpc.ab,ti.	9016
	3	exp prostate tumor/ OR exp Prostatic Neoplasms/ OR (((prostate OR prostatic) adj2 (neoplasm OR neoplasm$ OR cancer OR cancer$ OR carcinoma OR carcinoma$ OR adenocarcinoma OR adenocarcinoma$ OR tumour OR tumour$ OR tumor OR tumor$)).ab,ti.)	476,636
	4	(((castrate OR castration) adj3 resistan$) OR ‘hormone-refractory’ OR ‘hormone refractory’ OR ‘androgen-independent’ OR ‘androgen independent’).ab,ti.	43,929
	5	(advanced OR metastat$ OR refract$ OR recurren$ OR salva$ OR (‘late’ adj2 ‘stage’) OR resistan$ OR ‘stage iii’ OR (stage AND iii$) OR ‘stage iv’ OR ‘stage 3’ OR ‘stage 4’).ti,ab. OR (metastasis/ OR Neoplasm Metastasis/ OR exp Recurrence/ OR recurrent disease/)	6,604,276
	6	3 AND 4 AND 5	39,710
	7	1 AND 5	19,078

HRQOL Review			
	8	2 OR 6 OR 7	42,075
Outcomes: HRQoL	9	exp ‘quality adjusted life year’/ OR exp ‘health survey’/ OR exp ‘wellbeing’/ OR (exp ‘health’/ AND (state adj1 utilit$).ab,ti.)	365,191
	10	((utilit$ adj2 (measure$ OR outcome$ OR state$ OR health OR score$ OR weight$ OR analysis)) OR ‘health utility index‘ OR ‘hui‘ OR (utilit$ adj1 (score$ OR value$ OR evaluation$)) OR (health adj2 utilit$) OR (health AND (state adj1 utilit$)) OR ((health adj1 state$) AND (state$ adj1 preference$)) OR ‘quality adjusted life‘ OR (‘quality adjusted‘ adj1 survival$) OR qaly$ OR qald$ OR qale$ OR qtime$ OR ‘disability adjusted life‘ OR daly$ OR health survey OR hye OR health$year$equivalent OR (health adj2 utility$) OR wellbeing OR well-being OR (quality adj2 well$being) OR qwb OR (willingness adj2 pay) OR (standard adj2 gamble) OR disutili$ OR (time adj2 trade$off) OR tto OR (‘discrete choice‘ adj1 experiment$)).ti,ab.	672,434
	11	exp ‘quality of life‘/ OR exp ‘quality-of-life‘/ OR exp ‘short form 36‘ OR exp ‘short form 12‘/	570,967
	12	(‘quality of life’ OR ‘quality-of-life’ OR ‘qol‘ OR ‘hrqol‘ OR ‘hqol‘ OR ‘short form 36‘ OR ‘sf36‘ OR ‘sf-36‘ OR ‘sf 36‘ OR ‘short form 12‘ OR ‘sf12‘ OR ‘sf-12‘ OR ‘sf 12‘ OR ‘short form 6‘ OR ‘sf6‘ OR ‘sf-6‘ OR ‘sf 6‘ OR ‘euroqol‘ OR euro$qol OR ‘eq5d‘ OR ‘eq-5d‘ OR ‘eq 5d‘ OR rosser OR ((visual adj1 analog$) AND (analog$ adj1 scale$)) OR ‘European Organization for Research and Treatment of Cancer’ OR EORTC OR QLQ-C30 OR QLQ C30 OR QLQC30 OR QLQPR$ OR QLQ-PR$ OR ‘QLQ PR$’ OR ‘functional assessment of cancer therapy-prostate questionnaire‘ OR ‘fact-p‘ OR functional assessment of cancer therapy OR ‘FACT-G‘).ti,ab.	1,180,538
	13	or/9-12	2,062,698
**HRQoL studies in patients with mCRPC**	**14**	**8 AND 13**	**3851**

HRQOL Review			
**Irrelevant Study Design**	15	(addresses OR bibliography OR biography OR case report OR comment OR congresses OR consensus development conference OR duplicate publication OR editorial OR guideline OR *in vitro* OR interview OR lectures OR letter OR monograph OR news OR ‘newspaper article’ OR practice guideline OR ‘review literature’ OR ‘review of reported cases’ OR review, academic OR review, multicase OR review, tutorial OR twin study).pt.	4,396,415
	16	(animals/ not (humans/ and animals/)) OR (animal/ not (human/ and animal/))	6,050,706
	17	case report/ OR case reports/	4,894,359
	18	or/15-17	14,725,834
	19	14 not 18	3708
**Limit**	20	limit 19 to yr=‘2021 -Current’	281
**Final HRQoL studies in patients with mCRPC**	**21**	**Deduplicate**	**211**

ACP, American College of Physicians; EBM, Evidence-Based Medicine; HRQoL, health-related quality of life; mCRPC, metastatic castration-resistant prostate cancer; NHS, National Health Service; SLR, systematic literature review.

SUPPLEMENTAL TABLE S9

Economic SLR search strategy (updated systematic review)

**Economic Review**			
**Search conducted**	November 3, 2021		
**Databases searched**	EBM Reviews - Cochrane Database of Systematic Reviews <2005 to October 27, 2021><br>EBM Reviews - ACP Journal Club <1991 to October 2021><br>EBM Reviews - Database of Abstracts of Reviews of Effects <1st Quarter 2016><br>EBM Reviews - Cochrane Clinical Answers <October 2021><br>EBM Reviews - Cochrane Central Register of Controlled Trials <September 2021><br>EBM Reviews - Cochrane Methodology Register <3rd Quarter 2012><br>EBM Reviews - Health Technology Assessment <4th Quarter 2016><br>EBM Reviews - NHS Economic Evaluation Database <1st Quarter 2016><br>EconLit <1886 to October 21, 2021><br>Embase <1974 to 2021 November 02><br>Ovid MEDLINE(R) ALL <1946 to November 02, 2021>		
		**Term**	**Hits**
**Disease: mCRPC**	1	exp castration resistant prostate cancer/ OR exp Prostatic Neoplasms, Castration-Resistant/	21,026
	2	mcrpc.ab,ti.	9016
	3	exp prostate tumor/ OR exp Prostatic Neoplasms/ OR (((prostate OR prostatic) adj2 (neoplasm OR neoplasm$ OR cancer OR cancer$ OR carcinoma OR carcinoma$ OR adenocarcinoma OR adenocarcinoma$ OR tumour OR tumour$ OR tumor OR tumor$)).ab,ti.)	476,636
	4	(((castrate OR castration) adj3 resistan$) OR ‘hormone-refractory‘ OR ‘hormone refractory‘ OR ‘androgen-independent‘ OR ‘androgen independent‘).ab,ti.	43,929
	5	(advanced OR metastat$ OR refract$ OR recurren$ OR salva$ OR (‘late‘ adj2 ‘stage‘) OR resistan$ OR ‘stage iii‘ OR (stage AND iii$) OR ‘stage iv‘ OR ‘stage 3‘ OR ‘stage 4‘).ti,ab. OR (metastasis/ OR Neoplasm Metastasis/ OR exp Recurrence/ OR recurrent disease/)	6,604,276
	6	3 AND 4 AND 5	39,710
	7	1 AND 5	19,078
	8	2 OR 6 OR 7	42,075

Economic Review			
	9	exp ‘economics‘/ OR exp ‘economic aspect‘/ OR exp ‘cost‘/ OR exp ‘health care cost‘/ OR exp ‘drug cost‘/ OR exp ‘hospital cost‘/ OR exp ‘socioeconomics‘/ OR exp ‘health economics‘/ OR exp ‘pharmacoeconomics‘/ OR exp ‘fee‘/ OR exp ‘budget‘/ OR exp ‘hospital finance‘/ OR exp ‘financial management‘/ OR exp ‘health care financing‘/ OR exp ‘funding‘/ OR exp ‘finance‘/ OR exp ‘pricing‘/	1,836,472
	10	(cost$ OR (health$care adj1 cost$) OR (‘health care‘ adj1 cost$) OR fiscal OR funding OR financial OR finance OR financing OR (cost adj1 estimate$) OR ‘cost variable‘ OR (unit adj1 cost$) OR economic$ OR pharmacoeconomic$ OR socioeconomic$ OR price$ OR ‘pricing‘ OR (cost$ adj3 (treat$ OR therap$)) OR (health$care adj1 (utilisation OR utilization)) OR (‘health care‘ adj1 (utilisation OR utilization)) OR (resource adj1 (utilisation OR utilization OR ‘use’)) OR fee OR budget).ti,ab.	3,579,845
**Outcomes: Economic**	11	exp ‘economic evaluation’/ OR exp ‘cost effectiveness analysis’/ OR exp ‘cost benefit analysis’/ OR exp ‘cost efficiency analysis’/ OR exp ‘cost utility analysis’/ OR exp ‘monte carlo method’/	368,137
	12	((‘cost effectiveness’ adj3 analys$) OR (‘cost effectiveness’ adj3 model$) OR (‘cost effectiveness’ adj3 simulation$) OR (‘cost effective’ adj3 analys$) OR (‘cost effective’ adj3 model$) OR (‘cost effective’ adj3 simulation$) OR (‘cost effective’ adj3 assessment$) OR (‘cost benefit’ adj3 method$) OR (‘cost benefit’ adj3 analys$) OR (‘cost benefit’ adj3 model$) OR (‘cost benefit’ adj3 simulation$) OR (‘cost benefit’ adj3 assessment$) OR (‘cost efficiency’ adj3 analys$) OR (‘cost efficiency’ adj3 model$) OR (‘cost efficiency’ adj3 simulation$) OR (‘cost efficiency’ adj3 assessment$) OR (‘cost utility’ adj3 method$) OR (‘cost utility’ adj3 analys$) OR (‘cost utility’ adj3 model$) OR (‘cost utility’ adj3 simulation$) OR (‘cost utilities’ adj3 analys$) OR (‘cost utilities’ adj3 model$) OR (‘cost utilities’ adj3 simulation$) OR (‘cost utilities’ adj3 assessment$) OR (‘markov’ adj3 method$) OR (‘markov’ adj3 analys$) OR (‘markov’ adj3 model$) OR (‘markov’ adj3 simulation$) OR (‘markov’ adj3 assessment$) OR (markov adj3 analys$) OR (‘markov’ adj3 chain$) OR (‘monte carlo’ adj3 method$) OR (‘monte carlo’ adj3 analys$) OR (‘monte carlo’ adj3 model$) OR (‘monte carlo’ adj3 simulation$) OR (‘monte carlo’ adj3 chain$) OR (cost adj3 analys$) OR (cost adj3 model$) OR (cost adj3 simulation$) OR (cost adj3	306,715

Economic Review			
		assessment$) OR (economic adj3 analys$) OR (economic adj3 model$) OR (economic adj3 simulation$) OR (economic adj3 assessment$) OR ‘cost minimization analysis’ OR (‘cost minimization’ adj3 analys$) OR (‘cost minimization’ adj3 model$) OR (‘cost minimization’ adj3 simulation$) OR (‘cost minimization’ adj3 assessment$) OR ‘cost minimisation analysis’ OR (‘cost minimisation’ adj3 analys$) OR (‘cost minimisation’ adj3 model$) OR (‘cost minimisation’ adj3 simulation$) OR (‘cost minimisation’ adj3 assessment$) OR ‘budget impact analysis’ OR (‘budget impact’ adj3 analys$) OR (‘budget impact’ adj3 model$) OR (‘budget impact’ adj3 simulation$) OR (‘budget impact’ adj3 assessment$)).ti,ab.	
	13	or/9-12	4,835,297
**Economic outcomes in patients with mCRPC**	**14**	**8 AND 13**	**2313**
**Irrelevant Study Design**	15	(addresses OR bibliography OR biography OR case report OR comment OR congresses OR consensus development conference OR duplicate publication OR editorial OR guideline OR *in vitro* OR interview OR lectures OR letter OR monograph OR news OR ‘newspaper article’ OR practice guideline OR ‘review literature’ OR ‘review of reported cases’ OR review, academic OR review, multicase OR review, tutorial OR twin study).pt.	4,396,415
	16	(animals/ not (humans/ and animals/)) OR (animal/ not (human/ and animal/))	6,050,706
	17	case report/ OR case reports/	4,894,359
	18	or/15-17	14,725,834
	19	14 not 18	2230
**Limit**	20	limit 19 to yr=‘2021 -Current’	248
**Final Economic outcomes in patients**	**21**	**Deduplicate**	**200**

Economic Review			
**with mCRPC**			

ACP, American College of Physicians; EBM, Evidence-Based Medicine; mCRPC, metastatic castration-resistant prostate cancer; NHS, National Health Service; SLR, systematic literature review.

SUPPLEMENTAL TABLE S10

PICOS for original structured review (treatment patterns and safety)

Parameter	Medical background review[a]	Humanistic burden review[b]
Population	• Age: Adult men, i.e., ≥18 years of age<br>• Race: Any<br>• Disease: mCRPC	• Age: Adult men, i.e., ≥18 years of age<br>• Race: Any<br>• Disease: mCRPC
Study design	• Observational studies<br>• Retrospective studies<br>• Prospective studies<br>• Database registries<br>• Healthcare records<br>• Cohort analysis<br>• Longitudinal studies<br>• Case-control studies<br>• For diagnosis and prognosis reviews no restriction on study design was imposed	• Cohort studies<br>• Longitudinal studies<br>• Retrospective studies<br>• Prospective studies<br>• Case-control studies<br>• Cross-sectional studies<br>• Analysis of hospital records/database<br>• Single arm studies (uncontrolled trials)<br>• Registry studies
Line of therapy	• No restriction on line of therapy	
Intervention/comparator	• No restriction in terms of intervention or comparator	
Country	• No restriction in terms of country	
Language	• Articles published in English language were included	
Time frame	• Last 10 years (2009–2019)	

[a] Safety and treatment patterns.

[b] Health-related quality of life.

mCRPC: metastatic castration-resistant prostate cancer; PICOS: patient/population, intervention, comparison, outcome, and study design.

SUPPLEMENTAL TABLE S11

PICOS inclusion and exclusion criteria for updated interventional systematic review

Element	Inclusion		Exclusion	Rationale
Population	Adult males (≥18 years old) with pretreated, progressive mCRPC		Children and adolescents<br>Treatment-naïve patients with mCRPC	The population in whom the treatment is being appraised
Intervention	No restriction in terms of intervention or comparator		—	All treatments are being considered in this appraisal
Outcomes	**Efficacy**<br>• ORR<br>• CR/remission<br>• DoR<br>• PR/remission<br>• OS<br>• PFS<br>• Resistant disease<br>• Time to PSA progression<br>• Time to tumor progression<br>• Time to SSEs<br>• PSA response<br>• DCR<br>• Patients with SSEs<br>• Patients with tumor or PSA progression<br>• Time to first response<br>• Time to remission	**Safety/tolerability**<br>• AE (grade ≥3, all grades)<br>• Hypertension<br>• Diarrhea<br>• Nausea/Vomiting<br>• Fatigue<br>• Anorexia<br>• Peripheral edema<br>• Constipation<br>• Dehydration/hypotension<br>• Infection<br>• Arthralgia<br>• Decreased weight<br>• Urinary tract infection<br>• Thrombocytopenia<br>• Leukopenia<br>• Febrile neutropenia<br>• Abdominal pain<br>• Anemia<br>• Leukopenia<br>• Neurotoxicity<br>• Pain<br>• Bleeding<br>• Veno-occlusive disease	Studies not reporting any of the efficacy or safety outcomes of interest	These outcomes were evaluated in key trials for the treatment undergoing assessment

Element	Inclusion		Exclusion	Rationale
	• Progressive disease<br>• Time to treatment failure<br>• Stable disease<br>• Time to pain progression	• Death (30- and/or 60-day, induction death, treatment related, and overall)<br>• Discontinuations due to AEs		
Study design	RCTs (phase III)<br><br>*Reference lists of systematic literature reviews will be reviewed with a view of identifying any potential trial not captured through the database searches		Narrative reviews, editorials, commentary, letters, notes, short survey, case series or reports, animal or *in vitro* studies, open-label extensions, phase I trials, crossover studies without relevant data prior to crossover, observational studies	--
Language	English		Non-English publications	Most, if not all of the relevant evidence will be published in English

AE, adverse event; CR, complete response; DCR, disease control rate; mCRPC, metastatic castration-resistant prostate cancer; DoR, duration of response; ORR, objective response rate; OS, overall survival; PFS, progression-free survival; PICOS, patient/population, intervention, comparison, outcome, and study design; PR, partial response; PSA, prostate-specific antigen; RCT, randomized controlled trial; SSE, symptomatic skeletal event.

SUPPLEMENTAL TABLE S12

PICOS inclusion and exclusion criteria for updated HRQoL systematic review

Element	Inclusion	Exclusion	Rationale
Population	Adult males (≥18 years old) with pretreated, progressive mCRPC	Children and adolescents<br>Treatment-naïve patients with mCRPC	The population in whom the treatment is being appraised
Intervention	No restriction in terms of intervention or comparator	—	—
Outcomes	Original health state utility/disutility data, obtained using any methodology (e.g., TTO, SG, EQ-VAS, EQ-5D, SF-6D, HUI, QWB, or disease-specific utility instruments)<br>EORTC-QLQ-C30<br>Any other validated HRQoL measure	Studies not reporting any utility data or EORTC-QLQ-C30 outcomes	HRQoL outcomes are relevant to the reference case
Study design	RCTs, uncontrolled or nonrandomized interventional studies, single-arm clinical trials, observational studies	Narrative reviews, editorials, commentary, case series or reports, letters, animal or *in vitro* studies, pre/postindex period (or mirror image) studies	—
Temporal	From inception until September 4, 2020	—	—
Language	English	Non-English publications	Most, if not all of the relevant evidence will be published in English

EORTC-QLQ-C30, European Organization for Research and Treatment of Cancer Core Quality of Life questionnaire; EQ-5D, EuroQoL 5-dimension questionnaire; EQ-VAS, EuroQoL visual analog scale; HRQoL, health-related quality of life; HUI, Health Utility Index; mCRPC, metastatic castration-resistant prostate cancer; PICOS, patient/population, intervention, comparison, outcome and study design; QWB, Quality of Well-Being scale; RCT, randomized controlled trial; SF-6D, 6-dimension form of the Short-Form-36 measure; SG, standard gamble; TTO, time trade off.

SUPPLEMENTAL TABLE S13

PICOS inclusion and exclusion criteria for updated economic systematic review

Element	Inclusion	Exclusion	Rationale
Population	Adult males (≥18 years old) with pretreated, progressive mCRPC	Children and adolescents<br>Treatment-naïve patients with mCRPC	The population in whom the treatment is being appraised
Intervention	No restriction in terms of intervention or comparator	—	—
Outcomes	**Economic evaluations**<br>• Costs<br>• ICERs<br>• QALYs<br>• Life years<br>**Costs/HCRU**<br>• Total cost<br>• Direct and indirect cost components<br>• Healthcare resource use	**Economic evaluations**<br>• Studies reporting only cost and/or resource use<br>• Studies not reporting any economic outcomes of interest<br>**Costs/HCRU**<br>Data that are relevant to cost-effectiveness only	Data that are relevant to demonstrate the cost effectiveness and the costs/HCRU of the treatment being appraised
Study design	**Costs/resource use**<br>All study designs, including cost of illness studies, observational studies, retrospective database/registry studies or patient/medical chart reviews, HTAs reporting primary research, RCTs reporting resource use	Narrative reviews, editorials, commentary, letters, practice guidelines, case series or reports, animal or *in vitro* studies, open-label extensions, phase I trials	The study designs and publication types specified as eligible for inclusion were those considered most likely to report relevant data
Temporal	**Economic evaluations**<br>No restrictions<br>**Costs and HCRU**<br>January 2009 – current	**Economic evaluations**<br>No restrictions<br>**Costs and HCRU**<br>Not published within the search cut-off dates	**Economic evaluations**<br>No restrictions<br>**Costs and HCRU**<br>Cost/HCRU evidence are rapidly changing. Limiting the searches for this evidence to the

Element	Inclusion	Exclusion	Rationale
			last 10 years ensures that only the most recent, relevant data is identified
Language	English	Non-English publications	We anticipate that most, if not all of the relevant evidence will be published in English

HCRU, healthcare resource utilization; HTA, health technology assessment; ICER, incremental cost-effectiveness ratio; mCRPC, metastatic castration-resistant prostate cancer; PICOS, patient/population, intervention, comparison, outcome, and study design; QALY, quality-adjusted life year; RCT, randomized controlled trial.

SUPPLEMENTAL TABLE S14

PICOS for inclusion in the manuscript

**Parameter**	**Treatment patterns review**	**Humanistic burden review[a]**	**Interventional review**	**Economic review**
Population	Age: Adult men, i.e., ≥18 years of age<br>Race: Any<br>Disease: mCRPC<br>≥100 patients enrolled in the study			
Outcomes	No restriction			ICERs, QALYs, Total costs (including monthly total costs)
Study design	• Observational studies<br>• Retrospective studies<br>• Prospective studies<br>• Database registries<br>• Healthcare records<br>• Cohort analysis<br>• Longitudinal studies<br>• Case-control studies	• Cohort studies<br>• Longitudinal studies<br>• Retrospective studies<br>• Prospective studies<br>• Case-control studies<br>• Cross-sectional studies<br>• Analysis of hospital records/database<br>• Single arm studies (uncontrolled trials)<br>• Registry studies	For the updated review only phase III trials were included in the search strategy	• Cost-effectiveness analyses<br>• Budget impact models<br>• Health technology assessments,<br>• Cost-utility analyses<br>• Healthcare resource use

Parameter	Treatment patterns review	Humanistic burden review[a]	Interventional review	Economic review
		• Only studies which reported scores from validated PROs were included		• Only economic evaluations which reported ICERs and/or QALYs or studies that reported total costs were included
Intervention	2L+<br>Only approved/recommended[b] therapies in either comparator arm included			
Geographical	No restriction in terms of country			EU4, UK, and USA
Language	Articles published in English language were included			
Temporal	Last 5 years (2017–2021)			
Publication	Only journal articles, no congress abstracts			

[a] Health-related quality of life.

[b] At the time of the searches.

2L, second line; EU4, Germany, France, Spain, and Italy; ICER: incremental cost effectiveness ratio; mCRPC, metastatic castration-resistant prostate cancer; PICOS: patient/population, intervention, comparison, outcome, and study design; PRO: patient-reported outcome; QALY: quality-adjusted life year.

SUPPLEMENTAL TABLE S15

Summary of efficacy from pivotal trials of therapies used in clinical practice published prior to 2017

Study	N	Patient population	Intervention	Efficacy				
				mOS (95% CI), months	OS HR (95% CI)	mPFS (95% CI), months	PFS HR (95% CI)	ORR (95% CI) %
**TROPIC (1)**, **NCT00417079, global phase III trial**	378	Patients with mCRPC who are refractory to hormone therapy and previously treated with docetaxel-based chemotherapy	Cabazitaxel	15.1 (14.1–16.3)	0.70 (0.59–0.83) *P* < 0.0001	PFS 2.8 (2.4–3.0)[a]	0.74 (0.64–0.86) *P* < 0.0001	14.4 (9.6–19.3)[b]
	377		Mitoxantrone	12.7 (11.6–13.7)		PFS 1.4 (1.4–1.7)[a]		4.4 (1.6–7.2)[b]
**COU-AA-301 (2)**, **NCT00638690, global phase III trial**	797	Patients with mCRPC who were previously treated with docetaxel	Abiraterone + prednisone	15.8 (14.8–17.0)	0.74 (0.64–0.86) *P* < 0.0001	rPFS 5.6 (5.6–6.5)	0.66 (0.58–0.76) *P* < 0.0001	NR
	398		Placebo + prednisone	11.2 (10.4–13.1)		rPFS 3.6 (2.9–5.5)		NR
**AFFIRM (3)**, **NCT00974311, global phase III trial**	800	Patients with mCRPC who had previous treatment with docetaxel	Enzalutamide	18.4 (17.3–NR)	NR (NR–NR)	rPFS 8.3 (8.2–9.4)	0.4 (0.35–0.47) *P* < 0.001	29
	399		Placebo	13.6 (11.3–15.8)		rPFS 2.9 (2.8–3.4)		4

Study	N	Patient population	Intervention	Efficacy				
				mOS (95% CI), months	OS HR (95% CI)	mPFS (95% CI), months	PFS HR (95% CI)	ORR (95% CI) %
**IMPACT (4)**, **NCT00065442, phase III trial (USA and Canada)**	341	Patients with androgen-independent mCRPC who continued to receive androgen-deprivation therapy	Sipuleucel-T	NR	0.78 (0.61–0.98) *P* = 0.03	NR	NR	NR
	171		Placebo	NR		NR		
**ALSYMPCA (5)**, **NCT00699751, global phase III trial**	352[b]	Adult (age ≥18 years) with progressive mCRPC regardless of prior history of docetaxel	Ra-223 + best supportive care	14.4 (12.5–15.5)	0.70 (0.56–0.88) *P* = 0.002 (previous docetaxel use)[c]	NR	NR	NR
	174[b]		Placebo + best supportive care	11.3 (10.0–12.9)		NR		
**TAX-327 (6), global phase III trial (nonblinded)[d]**	335	Patients with progressive mHRPC	Docetaxel Q3W + prednisone	19.2 (17.5–21.3)	0.79 (0.67–0.93) *P* = 0.004 (vs. mitoxantrone)	NR	NR	NR
	334		Docetaxel QW + prednisone	17.8 (16.2–19.2)	0.87 (0.74–1.02) *P* = 0.086 (vs. mitoxantrone)	NR		
	337		Mitoxantrone Q3W + prednisone	16.3 (14.3–17.9)	Reference	NR		

[a] Composite PFS endpoint defined as time between randomization and the first date of progression as measured by PSA progression, tumor progression, pain progression, or death.

[b] Subgroup of patients who had received prior docetaxel.

[c] Similar data reported for previous docetaxel use and no previous docetaxel use.

[d] Final OS reported from Berthold et al. 2008 (7).

CI, confidence interval; HR, hazard ratio; mCRPC, metastatic castration-resistant prostate cancer; mHRPC, metastatic hormone-refractory prostate cancer; mOS, median overall survival; mPFS, median progression-free survival; NR, not reached; ORR, objective response rate; OS, overall survival; PFS, progression-free survival; PSA, prostate-specific antigen; Q3W, every 3 weeks; QW, once weekly; Ra-223, radium-223; rPFS, radiographic progression-free survival.

References

1. De Bono JS, Oudard S, Ozguroglu M, Hansen S, Machiels J-P, Kocak I, et al. Prednisone plus cabazitaxel or mitoxantrone for metastatic castration-resistant prostate cancer progressing after docetaxel treatment: a randomised open-label trial. *Lancet* (2010) 376(9747):1147-54. doi: 10.1016/S0140-6736(10)61389-X

2. Fizazi K, Scher HI, Molina A, Logothetis CJ, Chi KN, Jones RJ, et al. Abiraterone acetate for treatment of metastatic castration-resistant prostate cancer: final overall survival analysis of the COU-AA-301 randomised, double-blind, placebo-controlled phase 3 study. *Lancet Oncol* (2012) 13(10):983-92. doi: 10.1016/S1470-2045(12)70379-0

3. Scher HI, Fizazi K, Saad F, Taplin M-E, Sternberg CN, Miller K, et al. Increased survival with enzalutamide in prostate cancer after chemotherapy. *N Engl J Med* (2012) 367(13):1187-97. doi: 10.1056/NEJMoa1207506

4. Kantoff PW, Higano CS, Shore ND, Berger ER, Small EJ, Penson DF, et al. Sipuleucel-T immunotherapy for castration-resistant prostate cancer. *The New England journal of medicine* (2010) 363(5):411-22. doi: 10.1056/NEJMoa1001294

5. Hoskin P, Sartor O, O'sullivan JM, Johannessen DC, Helle SI, Logue J, et al. Efficacy and safety of radium-223 dichloride in patients with castration-resistant prostate cancer and symptomatic bone metastases, with or without previous docetaxel use: a prespecified subgroup analysis from the randomised, double-blind, phase 3 ALSYMPCA trial. *Lancet Oncol* (2014) 15(12):1397-406. doi: 10.1016/S1470-2045(14)70474-7

6. Tannock IF, De Wit R, Berry WR, Horti J, Pluzanska A, Chi KN, et al. Docetaxel plus prednisone or mitoxantrone plus prednisone for advanced prostate cancer. *The New England journal of medicine* (2004) 351(15):1502-12. doi: 10.1056/NEJMoa040720

7. Berthold DR, Pond GR, Soban F, De Wit R, Eisenberger M, Tannock IF. Docetaxel plus prednisone or mitoxantrone plus prednisone for advanced prostate cancer: updated survival in the TAX 327 study. *J Clin Oncol* (2008) 26(2):242-5. doi: 10.1200/JCO.2007.12.4008
